# Artificial Sweeteners and Autoimmune Diseases: Insights From Integrative Bioinformatics and Mendelian Randomization

**DOI:** 10.1002/fsn3.72032

**Published:** 2026-06-18

**Authors:** Yupei Liu, Jizhen Huang, Xue Hu, Shan Tian, Jiao Li, Shiyun Tan, Weiguo Dong

**Affiliations:** ^1^ Department of Gastroenterology Renmin Hospital of Wuhan University Wuhan China; ^2^ Department of Respiratory and Critical Care Medicine Renmin Hospital of Wuhan University Wuhan Hubei China; ^3^ Department of Oncology The General Hospital of Western Theater Command Chengdu China; ^4^ Department of Infection Union Hospital of Tongji Medical College of Huazhong University of Science and Technology Wuhan China

**Keywords:** artificial sweeteners, autoimmune diseases, environmental exposure, Mendelian randomization, network toxicology

## Abstract

Artificial sweeteners (AS) are widely used food additives, yet their potential role in autoimmune diseases (ADs) remains poorly understood. In this study, we integrated toxicological prediction, Mendelian randomization (MR), bioinformatics analyses, molecular docking, and gut microbiota assessment to investigate the association between AS exposure and ADs and to identify potential underlying mechanisms. Toxicity prediction using the ProTox‐II server indicated a high immunotoxic potential for AS (> 0.9). MR analysis revealed a positive association between artificially sweetened cereal consumption and ADs risk (OR = 1.223, 95% CI: 1.005–1.488, *p* = 0.04). A total of 209 AS‐related genes (ARGs) were identified through target prediction databases. Functional enrichment analyses demonstrated that these genes were primarily involved in apoptosis, TNF signaling, and IL‐17 signaling pathways, suggesting their contribution to immune dysregulation. By integrating maximal clique centrality (MCC) algorithms with MR evidence, six core targets, including EPHX2, ESR1, ITGB3, MMP9, IL2RA, and PIM1, were identified as potential mediators of AS‐associated ADs. Molecular docking further supported the binding affinity between AS compounds and these target proteins. In addition, gut microbiota analysis suggested that AS may suppress beneficial bacteria such as *Akkermansia muciniphila* , thereby disrupting arachidonic acid metabolism and chemokine signaling pathways involved in autoimmune pathogenesis. Collectively, these findings establish a multi‐omics framework linking AS exposure to autoimmune dysregulation and provide novel insights into the immunotoxicological effects of AS and their potential role in the development of ADs.

## Graphical abstract

This study provides a novel molecular perspective on the role of AS in ADs development. We identified a positive association between artificially sweetened cereal intake and ADs risk and revealed multiple pathogenic mechanisms involving apoptosis, inflammatory signaling, immune activation, and gut microbiota dysbiosis. Furthermore, EPHX2, ESR1, ITGB3, MMP9, IL2RA, and PIM1 were identified as core targets potentially mediating the immunotoxic effects of AS. Our findings suggest that reducing AS exposure may represent a practical dietary strategy for preventing autoimmune dysregulation. These results provide important evidence linking food additive exposure to autoimmune disease susceptibility and support the reassessment of artificial sweetener safety in vulnerable populations.

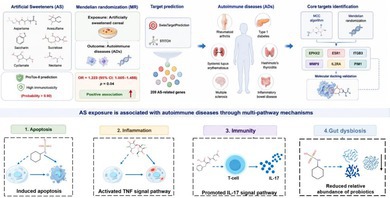

## 1 Introduction

Artificial sweeteners (AS) refer to a class of chemical substances used as substitutes for sucrose (common sugar) to provide sweetness in foods and beverages. Compared with sucrose, AS not only contain little or no caloric value but also exhibit markedly higher sweetness, in some cases hundreds of times greater than that of sucrose. Common AS include aspartame, saccharin, sucralose, and acesulfame potassium (acesulfame K), among others (Group and Group 2023). In recent years, the global prevalence of chronic diseases such as obesity, diabetes, and cardiovascular disorders has continued to rise, accompanied by a substantial increase in public awareness of dietary health (Collaborators 2025; Global Burden of Cardiovascular Diseases and Risks 2023 Collaborators 2025). Excessive sucrose consumption is widely recognized as a major risk factor for obesity, diabetes, and related metabolic complications. Consequently, AS, which satisfy sweetness preferences without adding caloric burden, have become popular alternatives to sucrose (Wilk et al. 2022). In particular, for individuals requiring weight management or those at risk of diabetes, the properties of AS align well with the demand for sugar‐free or low‐sugar food products (Debras et al. 2023), thereby driving sustained growth in the consumption of low‐calorie beverages and snacks (Chen et al. 2023). Despite their widespread use, the safety of AS has long remained a subject of public and academic debate. Regulatory authorities such as the World Health Organization (WHO) and the U.S. Food and Drug Administration (FDA) have concluded, based on toxicological evaluations, that commonly used AS are safe when consumed within established acceptable daily intake levels (Group and Group 2023). Nevertheless, an increasing body of evidence suggests that long‐term or excessive AS consumption may be associated with adverse health outcomes.

Autoimmune diseases (ADs) are a group of chronic inflammatory disorders caused by the breakdown of immune tolerance, characterized by the immune system mistakenly recognizing self‐antigens and triggering persistent tissue damage (Ruggeri et al. 2025). Rheumatoid arthritis (RA), multiple sclerosis (MS), inflammatory bowel disease (IBD), type 1 diabetes (T1D), and Hashimoto's thyroiditis (HT) are representative ADs. Although the clinical manifestations of ADs vary considerably, their pathogenesis appears to involve common mechanisms, including abnormal activation of Th1/Th17 cells, impaired regulatory T cell (Treg) function, persistent release of pro‐inflammatory cytokines such as IL‐6, TNF‐α, and IFN‐γ, as well as gut microbiota dysbiosis (Fu et al. 2025; Park and Ciofani 2025). Notably, increasing evidence indicates that AS may contribute to the immune dysregulation associated with ADs. Epidemiological studies have reported that frequent consumption of artificially sweetened beverages is associated with increased inflammatory markers, metabolic syndrome, and a higher risk of immune‐related diseases such as RA (Ascione et al. 2023; Jamali et al. 2025). Animal studies further demonstrated that long‐term intake of sucralose or saccharin can alter gut microbiota composition, reduce short‐chain fatty acid production, and promote chronic low‐grade inflammation (Del Pozo et al. 2022). Importantly, Zani et al. reported that high‐dose sucralose suppressed T‐cell proliferation and differentiation, impaired T‐cell receptor (TCR) signaling and calcium mobilization, and consequently altered T cell‐mediated immune responses (Zani et al. 2023). Subsequent work by Kränkel et al. suggested that these effects may be mediated through alterations in membrane lipid structure, oxidative stress, and disruption of the gut–immune axis, potentially contributing to the progression of T cell‐dependent ADs such as MS, T1D, and IBD (Krankel and Rauch‐Kroehnert 2023).

Although existing studies suggest that AS are associated with metabolic disorders, gut microbiota dysbiosis, and immune‐inflammatory responses, current studies investigating the relationship between AS and ADs remain limited. First, most existing studies on the immunotoxic effects of AS are based on epidemiological associations or retrospective analyses, which are susceptible to multiple confounding factors (Dan et al. 2023). Second, experimental studies have largely focused on individual sweeteners, single diseases, or localized inflammatory models, with primary attention given to metabolic toxicity and gut‐related effects (Wu et al. 2024; Yang et al. 2026; Zhong et al. 2024). Consequently, the genetic causal relationship between AS exposure and ADs, as well as the shared immune‐inflammatory pathways and key targets through which AS may contribute to the development and progression of different ADs, have not been systematically elucidated. Therefore, this study aimed to comprehensively investigate the molecular and immunological mechanisms underlying the associations between AS exposure and ADs by integrating Mendelian randomization (MR), network toxicology, molecular docking, and gut microbiota‐based analyses, thereby providing novel insights into the potential mechanisms underlying AS‐associated immune dysregulation.

## 2 Methods

### 2.1 Toxicological Analysis of AS

Toxicity predictions for the compound structural models of seven AS were conducted using the ProTox‐II databases to obtain relevant information regarding their induced toxicity (Banerjee et al. 2018). The SMILES sequences for AS were sourced from PubChem, SwissTargetPrediction, and STITCH database.

### 2.2 Two‐Sample Mendelian Randomization

#### 2.2.1 Data Sources

To evaluate the overall effects of AS on ADs, a two‐sample MR analysis was conducted. Genetic instruments for AS intake were obtained from the IEU OpenGWAS project (https://gwas.mrcieu.ac.uk/, accessed May 20, 2026). Three AS‐related traits were selected as exposures: artificial sweetener added to cereals intake (ukb‐b‐3143), artificial sweetener added to coffee intake (ukb‐b‐1338), and artificial sweetener added to tea intake (ukb‐b‐5867). Instrumental variables (IVs) were selected through sequential criteria (Sekula et al. 2016): (1) Single nucleotide polymorphisms (SNPs) associated with exposures at genome‐wide significance (*p* < 5 × 10^−8^) were initially selected. Due to the limited number of eligible SNPs, the threshold was relaxed to *p* < 5 × 10^−6^. (2) The IVs must be free from confounding influences that could affect the outcome, thereby supporting accurate causal inference. Linkage disequilibrium (LD) clumping was subsequently performed using an *r* ^2^ threshold of 0.001 and a clumping distance of 10,000 kb to ensure independence among selected SNPs. (3) The IVs should influence the outcome only through their effect on the exposure variable, which helps to eliminate biases arising from pleiotropy. In addition, SNPs with *F*‐statistics < 10 were removed to avoid weak instrument bias, and the *F*‐value was calculated according to the following equation:

$$F=\left(\frac{n-k-1}{2k}\right)\times \left(\frac{R^{2}}{1-R^{2}}\right)$$

Outcome data for ADs were obtained from the FinnGen Consortium R12 (https://www.finngen.fi/en) release. Both exposure and outcome datasets were derived from European populations (Tu et al. 2024). Prior to MR analysis, harmonization procedures were performed to align exposure and outcome alleles and ensure that SNP effects corresponded to the same effect allele. To satisfy the third MR assumption, SNPs showing stronger associations with the outcome than with the exposure, or directly associated with the outcome (*p* < 0.05), were excluded. Detailed information on exposure and outcome datasets is provided in Tables S1 and S2.

#### 2.2.2 Mendelian Randomization Analysis

For single‐SNP instrumental variables, causal effects were estimated using the Wald ratio method (Bowden et al. 2016). For analyses involving multiple SNPs, five MR methods were applied, including inverse variance weighted (IVW), MR‐Egger, weighted median, simple mode, and weighted mode approaches. The IVW fixed‐effects model was used as the primary method for causal inference, while MR‐Egger, weighted median, simple mode, and weighted mode analyses were performed as complementary approaches to assess the robustness of the results.

#### 2.2.3 Sensitivity Analysis

Sensitivity analyses were conducted to evaluate the reliability and robustness of the MR results, including Cochran's *Q* test, MR‐Egger intercept analysis, and leave‐one‐out analysis (Verbanck et al. 2018). Cochran's Q test was used to assess heterogeneity among SNPs. A fixed‐effects IVW model was applied when *p* > 0.05; otherwise, a random‐effects IVW model was used. Horizontal pleiotropy was evaluated using the MR‐Egger intercept test, where *p* > 0.05 indicated no evidence of significant pleiotropic bias. Leave‐one‐out analysis was further performed to determine whether the overall causal estimates were driven by any individual SNP (Bowden et al. 2015).

#### 2.2.4 Statistical Analysis

Statistical significance was defined as *p* < 0.05, and causal effects were reported as odds ratios (ORs) with 95% confidence intervals (CIs). To account for multiple testing, Benjamini–Hochberg false discovery rate (FDR) correction was applied (Ma, Qi, et al. 2025). Associations with *Pfdr* < 0.05 were considered to provide strong evidence for causality, whereas associations with 0.05 ≤ *Pfdr* < 0.20 were regarded as suggestive evidence. All analyses were performed using R software (version 4.4.3) and the TwoSampleMR package (v0.5.6).

### 2.3 Acquisition of AS Targets

A comprehensive target identification strategy was employed to construct a reliable AS‐related target database. Seven commonly used AS were selected based on their widespread consumption in China (Wang et al. 2021), the United States (U.S. Food and Drug Administration 2014), the European Union and other countries (Samaniego‐Vaesken et al. 2018), including aspartame, acesulfame‐K, sucralose, neohesperidin dihydrochalcone (NHDC), sodium cyclamate, neotame, and saccharin. Detailed information on these compounds is provided in Table S3. The chemical structures and canonical SMILES representations of the selected AS were retrieved from the PubChem database (Kim et al. 2025). These structural data are essential for subsequent target prediction analyses. Potential protein targets of the seven AS were then predicted using the SwissTargetPrediction (http://www.swisstargetprediction.ch) and STITCH (https://stitch‐db.org/) databases based on their corresponding SMILES representations (Daina et al. 2019; Szklarczyk et al. 2016). The species was restricted to *Homo sapiens* , and a probability threshold of > 0.01 was applied in SwissTargetPrediction (Han et al. 2025). Finally, targets obtained from the two databases were merged to generate a nonredundant target list for each AS. The individual target sets of all seven sweeteners were subsequently combined to obtain the collective AS‐associated target pool for downstream analyses.

### 2.4 Acquisition of Autoimmune Disease Related Targets

In the present study, AD‐related datasets were obtained from the Gene Expression Omnibus (Jian et al. 2025) (GEO; https://www.ncbi.nlm.nih.gov/geo/) and the GeneCards database (Stelzer et al. 2016) (version 5.26); https://www.genecards.org/: (1) From GEO, AD‐related datasets were downloaded to identify AD‐associated differentially expressed genes (DEGs). For data normalization, log2 transformation was applied, and individual batch effects were eliminated via the removeBatchEffect in the limma R package (Figure S1). After data normalization, the datasets were imported into R software, and differential expression analysis between disease groups and normal controls was performed using the limma package. DEGs were screened according to the criteria of |log_2_ fold change (FC)| > 0.3 and *p* < 0.05, resulting in a set of AD‐related DEGs. Detailed information on the GEO datasets included in this study is provided in Table S4. (2) From the GeneCards database, genes associated with six ADs—RA, SLE, MS, IBD, HT, and T1D—were retrieved. To enhance the relevance of the identified genes, disease‐associated genes were filtered using the median correlation score as the threshold (Xin et al. 2025). Finally, AD‐related genes obtained from GEO and GeneCards were merged and deduplicated to retain unique targets. These targets were subsequently intersected with AS‐related genes (ARGs) to identify AS‐associated genes for each AD (AARGs), which served as the basis for downstream analyses.

### 2.5 Construction and Functional Analysis of Protein–Protein Interaction Networks

To explore the functional relationships between AS‐related targets and AD‐associated genes, protein–protein interaction (PPI) networks were constructed using the STRING database (version 12.0; https://string‐db.org/). *Homo sapiens* was selected as the organism, interaction sources were limited to “experiments” and “databases,” and the minimum required interaction score was set to 0.4 (Szklarczyk et al. 2021). Functional enrichment analyses were conducted for Gene Ontology (GO) terms, including biological processes (BP), molecular functions (MF), and cellular components (CC), as well as Kyoto Encyclopedia of Genes and Genomes (KEGG) pathways. Enrichment analyses were performed using the clusterProfiler package (version 3.14.3) via hypergeometric testing, with parameters set as minGSSize = 10 and maxGSSize = 500. The Benjamini–Hochberg method was applied for multiple testing correction. Terms with raw *p* < 0.05 and a FDR < 0.25 were considered statistically significant (Kanehisa et al. 2025). Visualization of enrichment results was performed using the ggplot2 package (version 3.4.2).

### 2.6 Mendelian Randomization Analysis Between Hub ARGs and ADs

#### 2.6.1 Data Sources

Cis‐expression quantitative trait loci (cis‐eQTL) data for all ARGs were obtained from the eQTLGen Consortium (Phase I, https://eqtlgen.org/cis‐eqtls.html). The eQTLGen dataset includes cis‐eQTL data for 15,695 genes derived from blood samples of 31,684 participants from multiple European cohorts, enabling the assessment of regulatory relationships between genetic variants and gene expression.

Outcome data for each AD were obtained from the FinnGen Consortium R12 (https://www.finngen.fi/en) release. Detailed information regarding exposure and outcome datasets is provided in Table S1.

#### 2.6.2 Instrumental Variable Selection

SNPs associated with cis‐eQTLs of overlapping target genes were selected as IVs according to the following criteria (Gkatzionis et al. 2023): (1) genome‐wide significance threshold of *p* < 5.0 × 10^−8^ to ensure robust associations between SNPs and gene expression while minimizing false‐positive results; (2) linkage disequilibrium (LD) pruning with an r (Collaborators 2025) threshold < 0.1 to reduce the effects of multicollinearity. When the number of eligible SNPs was limited, the threshold was relaxed to r² < 0.3; and (3) a clumping window of 10,000 kb to ensure proximity between selected SNPs and target genes, thereby improving the reliability of SNPs as regulators of gene expression. To evaluate weak instrument bias, *F*‐statistics were calculated to assess instrument strength. SNPs with *F*‐statistics < 10 were considered weak instruments and were excluded from subsequent analyses (Table S5) (Hartwig et al. 2017).

#### 2.6.3 Mendelian Randomization Analysis

The MR procedures, sensitivity analyses, and statistical criteria were performed as described in Sections 2.2.2, 2.2.4.

### 2.7 Hub Gene Screening

Hub targets were identified through an integrative strategy combining the maximal clique centrality (MCC) algorithm in CytoHubba with MR analysis. This approach enabled the simultaneous screening of core targets from both network topological significance and genetically predicted disease association perspectives, thereby enhancing the reliability and robustness of the findings. Selecting the MCC algorithm was based on its superior sensitivity and accuracy in predicting essential proteins within biological networks, particularly its ability to effectively identify key nodes located in densely connected interaction modules (Chin et al. 2014; Zheng et al. 2026). After importing the protein–protein interaction (PPI) network into Cytoscape software, the top 10 genes ranked by the MCC algorithm in the CytoHubba plugin were defined as potential hub genes. These genes were subsequently intersected with AD‐associated risk genes identified through MR analysis to obtain AS‐related hub genes potentially involved in the pathogenesis of each AD.

### 2.8 Molecular Docking Between AS and Core Targets

Molecular docking analyses were performed to evaluate the binding potential between AS compounds and core target proteins. Crystal structures of target proteins were obtained from the RCSB Protein Data Bank (https://www.rcsb.org/) using the following criteria: *Homo sapiens* origin, resolution ≤ 3.0 Å, and sequence identity ≥ 90% (Bittrich et al. 2024). Protein structures were preprocessed using PyMOL (version 3.1.4) by removing water molecules and co‐crystallized ligands. Ligand structures of AS compounds were retrieved from the PubChem database. Docking simulations were conducted using the CB‐Dock2 platform (http://clab.labshare.cn/cb‐dock2/), which integrates cavity detection and template‐based docking strategies (Liu et al. 2022). Blind docking was initially performed using AutoDock Vina with the following parameters: exhaustiveness = 32, maximum poses = 20, energy range = 5 kcal/mol, RMSD cutoff = 1.0 Å, and docking box size automatically adjusted to 1.5‐fold of the predicted cavity volume. Template‐based docking using FitDock was additionally applied when suitable homologous complexes were available. Binding affinity was evaluated using consensus scoring integrating Vina and FitDock energy functions, and the conformation with the lowest binding energy was selected as the optimal docking pose for subsequent analyses.

### 2.9 Gut Microbiome Analysis

Gut microbiota analysis was performed to identify potential microbial targets involved in ARGs‐induced ADs pathogenesis. Host gene–microbe associations were retrieved from the Gut Microbe–Gene database (gutMGene; http://www.gutmgene.org/; accessed December 27, 2025), with filters applied for human host species (Qi et al. 2025). Microbial taxa sharing target genes with AS were identified, and associated metabolites were subsequently retrieved with constraints including literature‐supported evidence and available PubChem CIDs. Potential protein targets of these metabolites were predicted using the Similarity Ensemble Approach (Gu and Lai 2020) (SEA: https://sea.bkslab.org/) and ChEMBL databases (https://www.ebi.ac.uk/chembl/). GO and KEGG enrichment analyses were conducted to elucidate biological pathways involved in microbiota‐mediated ADs modulation. Finally, an integrated interaction network linking AS, host targets, gut microbiota, microbial metabolites, and downstream host genes was constructed using Cytoscape to systematically illustrate the multi‐component regulatory framework underlying ARGs‐induced ADs pathogenesis.

## 3 Results

### 3.1 Toxicity Prediction of AS and Their Causal Associations With ADs

Given the widespread use of AS in daily life (Figure S2A,B), the ProTox‐II database was used to predict the potential toxicity of six AS compounds. The results showed that, except for sucralose with an immunotoxicity probability of 0.68, the remaining five AS compounds exhibited high predicted immunotoxicity probabilities exceeding 0.90 (Figure S2C). In addition, the toxicity prediction suggested potential cardiotoxicity, carcinogenicity, hepatotoxicity, and neurotoxicity for these AS compounds.

Based on these findings, two‐sample MR analyses were further performed to investigate the associations between three AS‐related dietary exposures, including intake of cereals, coffee, and tea containing added artificial sweeteners, and the risk of ADs. IVW analysis demonstrated that intake of cereals containing added AS was associated with an increased risk of ADs (OR = 1.223, 95% CI: 1.005–1.488, *p* = 0.04), whereas consumption of coffee (OR = 1.000, 95% CI: 0.839–1.190, *p* = 0.997) or tea (OR = 0.992, 95% CI: 0.848–1.160, *p* = 0.917) containing added AS showed no significant association with AD risk. Moreover, the weighted median, simple mode, and weighted mode methods showed effect estimates consistent with the direction of the IVW results for cereals containing added artificial sweeteners, although statistical significance was not observed (Table 1). Scatter plots and funnel plots further visualized these findings, while sensitivity analyses additionally supported the robustness of the results (Figure S2D–F).

**TABLE 1:** The genetic causal association between ASs and autoimmune diseases tested by the MR study.

Exposure‐outcome	Method	SNPs	OR (95% CI)	*p*	*Q*	*Q*_df	*Q*_*p*	Egger_intercept	SE	*p*
Intake of AS added to cereal‐AD	MR Egger	20	1.293 (0.892–1.874)	0.192	9.568	18	0.945	−0.001	0.003	0.735
	Weighted median	20	1.279 (0.983–1.665)	0.067	9.687	19	0.960			
	Inverse variance weighted	20	1.223 (1.005–1.488)	0.044						
	Simple mode	20	1.35 (0.854–2.134)	0.214						
	Weighted mode	20	1.31 (0.9–1.907)	0.175						
Intake of AS added to coffee‐AD	MR Egger	14	1.097 (0.791–1.52)	0.59	20.286	12	0.06	−0.004	0.005	0.519
	Weighted median	14	1.025 (0.845–1.244)	0.799	21.032	13	0.07			
	Inverse variance weighted	14	1 (0.839–1.19)	0.997						
	Simple mode	14	1.04 (0.79–1.368)	0.786						
	Weighted mode	14	0.992 (0.809–1.217)	0.942						
Intake of AS added to tea‐AD	MR Egger	17	1.17 (0.835–1.639)	0.375	19.181	15	0.206	−0.005	0.005	0.295
	Weighted median	17	0.929 (0.765–1.129)	0.461	20.687	16	0.191			
	Inverse variance weighted	17	0.992 (0.848–1.16)	0.917						
	Simple mode	17	0.967 (0.708–1.321)	0.836						
	Weighted mode	17	0.942 (0.744–1.192)	0.624						

### 3.2 Acquisition and Functional Enrichment of Artificial Sweetener Related‐Targets

To further investigate the key targets through which AS may contribute to the development and progression of ADs, network toxicology analysis was performed to identify ARGs for subsequent analyses. The SMILES strings of seven commonly used AS were submitted to the SwissTargetPrediction and STITCH databases to predict their potential molecular targets. Based on the predefined screening criteria, 92 targets were identified for aspartame, 12 for acesulfame, 25 for sucralose, 15 for NHDC, 13 for cyclamate, 102 for neotame, and 23 for saccharin. After removing duplicate entries, a total of 209 unique ARGs were obtained (Table S6). These targets were subsequently imported into the STRING database to construct a PPI network. After applying a high‐confidence interaction threshold and removing isolated nodes, 206 targets remained, forming a tightly interconnected network comprising 429 edges (Figure S2G). Functional enrichment analysis revealed that these targets were primarily enriched in BP related to regulation of inflammatory response, amyloid‐beta metabolic process, and adenylate cyclase–modulating G protein–coupled receptor signaling pathways. MF analysis indicated significant enrichment in endopeptidase activity, carbonate dehydratase activity, and peptide receptor activity, whereas CC analysis showed predominant localization in membrane rafts and presynaptic membranes (Figure S2H). KEGG pathway enrichment further suggested that AS may contribute to the initiation and progression of disease mainly by modulating inflammation and metabolism related pathways (Figure S2I). Using the Degree algorithm in the CytoHubba plugin, CASP3, MMP9, IL1B, SRC, MAPK1, DPP4, XIAP, GCG, CASP8, and ITGB1 were identified as the core functional targets potentially regulated by ARGs.

### 3.3 Biological Effects and Identification of Core ARGs in Rheumatoid Arthritis

**FIGURE 1:**
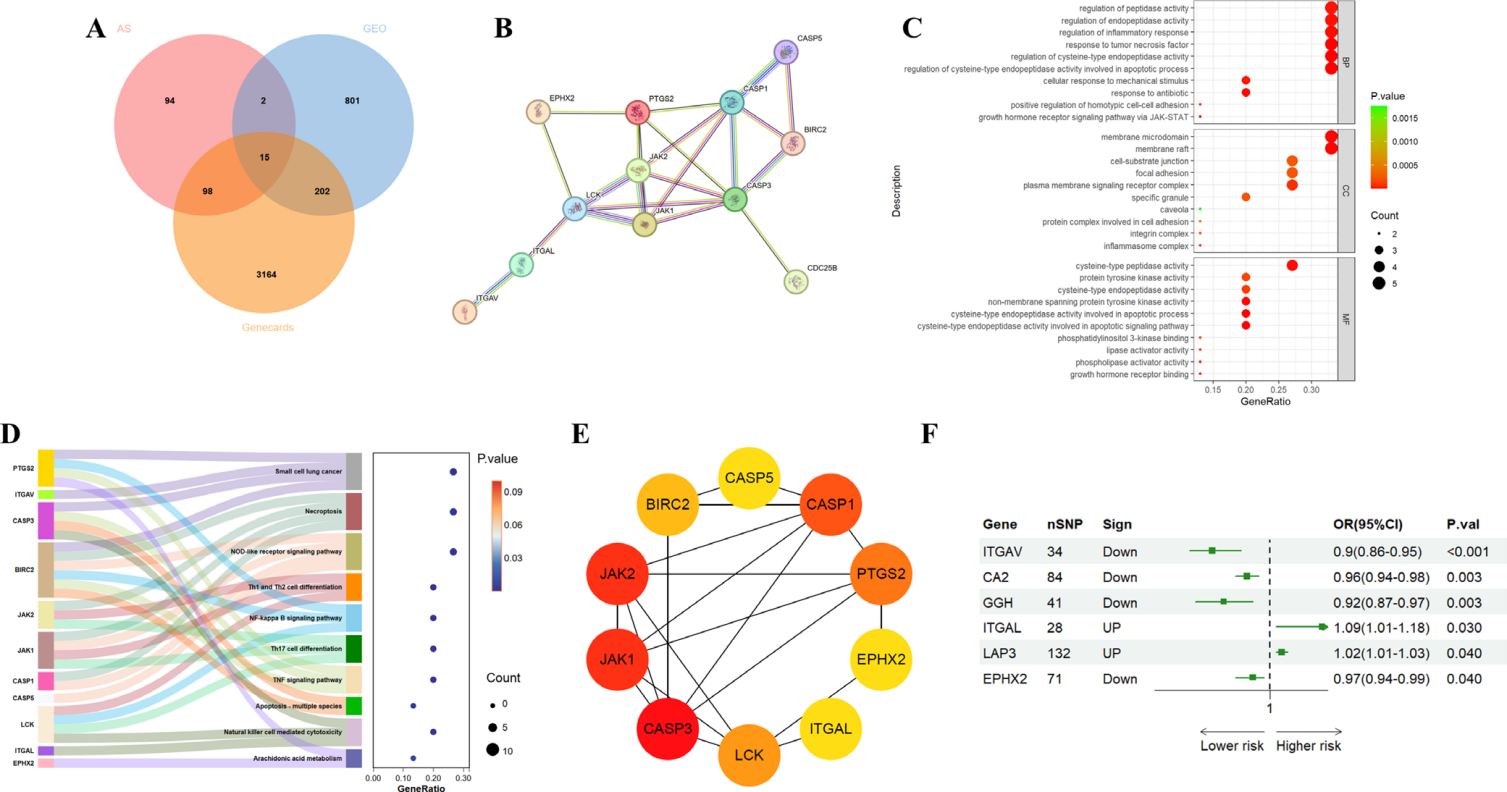
Integrated analysis screening the AS‐associated molecular networks and functional enrichment in Rheumatoid arthritis. (A) Venn diagram of AS targets overlapping in GeneCards and GEO dataset. (B) PPI network of core overlapping targets. (C) GO enrichment analysis of the overlapping target genes identified, divided into three categories: Including BP, CC, and MF. (D) KEGG enrichment analysis showed the top 10 pathways with the highest count values and identified key genes in these 10 pathways. (E) The top 10 core targets with the highest scores identified by MCC algorithm. (F) MR analysis screening of core targets in overlapping targets. The *x*‐axis represents the odds ratio (OR), with the vertical line indicating the null effect line (OR = 1). Green Square represent the OR values obtained from different MR analysis methods, with horizontal lines denoting the 95% confidence intervals (CI).

A total of 3479 and 1020 RA‐related genes were retrieved from the GeneCards and GEO databases, respectively. Intersection analysis between RA‐related genes and ARGs identified 15 overlapping genes (Table S7), which were considered potential ARGS‐induced RA‐associated targets (Figure 1A,B). Functional enrichment analysis of these 15 targets was mainly associated with BP such as regulation of cysteine‐type endopeptidase activity, response to tumor necrosis factor, and regulation of inflammatory response. MF analysis showed enrichment in cysteine‐type peptidase activity, lipase activator activity, and phospholipase activator activity, while CC analysis suggested predominant localization in membrane rafts, membrane microdomains, and plasma membrane signaling receptor complexes (Figure 1C). KEGG pathway analysis revealed significant enrichment in the TNF signaling pathway, Th1 and Th2 cell differentiation, and arachidonic acid metabolism (Figure 1D). To further identify core ARGs in RA, the MCC algorithm was applied to identify CASP1, CASP3, CASP5, PTGS2, EPHX2, ITGAL, LCK, JAK1, JAK2, and BIRC2 as hub genes (Figure 1E; Table S8). Subsequently, two‐sample MR analysis demonstrated that six of the 15 overlapping genes exhibited statistically significant causal associations with RA progression. Among these, ITGAL and LAP3 were identified as risk factors, whereas ITGAV, CA2, GGH, and EPHX2 were identified as protective factors. However, ITGAL, ITGAV, and GGH were excluded due to inconsistencies between differential expression analysis and MR results (Figure 1F). By integrating MCC and MR analyses, EPHX2 was ultimately identified as a robust candidate target mediating ARGs‐induced RA. Further MR analysis specifically targeting EPHX2 demonstrated that EPHX2 acted as a protective factor against RA risk and the sensitivity analyses further supported the robustness of this finding (Figure S3A; Table S9).

### 3.4 Biological Effects and Identification of Core ARGs in Systemic Lupus Erythematosus

**FIGURE 2:**
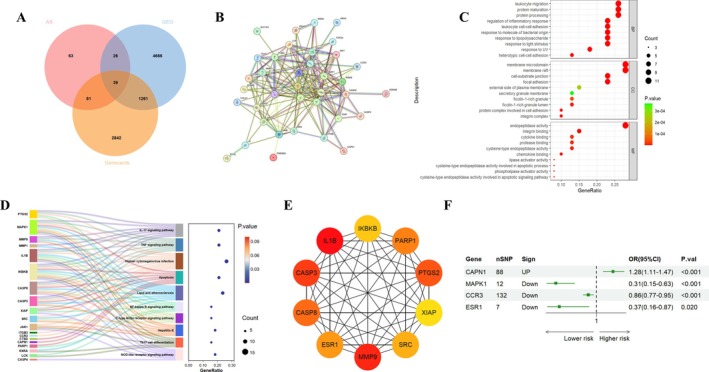
Integrated analysis screening the AS‐associated molecular networks and functional enrichment in systemic lupus erythematosus. (A) Venn diagram of AS targets overlapping in GeneCards and GEO dataset. (B) PPI network of core overlapping targets. (C) GO enrichment analysis of the overlapping target genes identified, divided into three categories: including BP, CC, and MF. (D) KEGG enrichment analysis showed the top 10 pathways with the highest count values and identified key genes in these 10 pathways. (E) The top 10 core targets with the highest scores identified by MCC algorithm. (F) MR analysis screening of core targets in overlapping targets. The *x‐ax*is represents the odds ratio (OR), with the vertical line indicating the null effect line (OR = 1). Green square represents the OR values obtained from different MR analysis methods, with horizontal lines denoting the 95% confidence intervals (CI).

A total of 4223 and 5992 SLE‐related genes were selected from the GeneCards and GEO databases, respectively. Intersection analysis between SLE‐related genes and ARGs identified 39 overlapping genes (Table S7), which formed a densely connected PPI network (Figure 2A,B). Functional enrichment analysis of these 39 targets indicated that ARGs‐induced SLE mechanisms were mainly associated with BP such as protein processing, leukocyte migration, and regulation of inflammatory response. MF analysis showed enrichment in endopeptidase activity, chemokine binding, and lipase activator activity, while CC analysis suggested predominant localization in membrane rafts, focal adhesions, and plasma membrane signaling receptor complexes (Figure 2C). KEGG pathway analysis revealed significant enrichment in the IL‐17 signaling pathway, TNF signaling pathway, and apoptosis (Figure 2D).

The MCC algorithm identified IL1B, MMP9, CASP3, PTGS2, CASP8, PARP1, ESR1, SRC, IKBKB, and XIAP as hub genes (Figure 2E; Table S8). Subsequently, two‐sample MR analysis demonstrated that CASP3, IKBKB, ITGAL, CAPN1, MMP1, MAPK1, PARP1, CCR3, DPP4, ELANE, ESR1, and ITGA4 exhibited statistically significant causal associations with SLE progression. After excluding genes with inconsistent differential expression analysis results or significant horizontal pleiotropy, CAPN1, CCR3, MAPK1, and ESR1 were identified as key ARGs associated with SLE in the MR analysis (Figure 2F). By integrating MCC and MR analyses, ESR1 was ultimately identified as a robust candidate target mediating ARGs‐induced SLE. Further MR analysis focusing on ESR1 indicated that increased genetically predicted ESR1 expression was associated with a reduced risk of SLE, and the sensitivity analyses further confirmed the stability and reliability of this association (Figure S3B; Table S9).

### 3.5 Biological Effects and Identification of Core ARGs in Multiple Sclerosis

**FIGURE 3:**
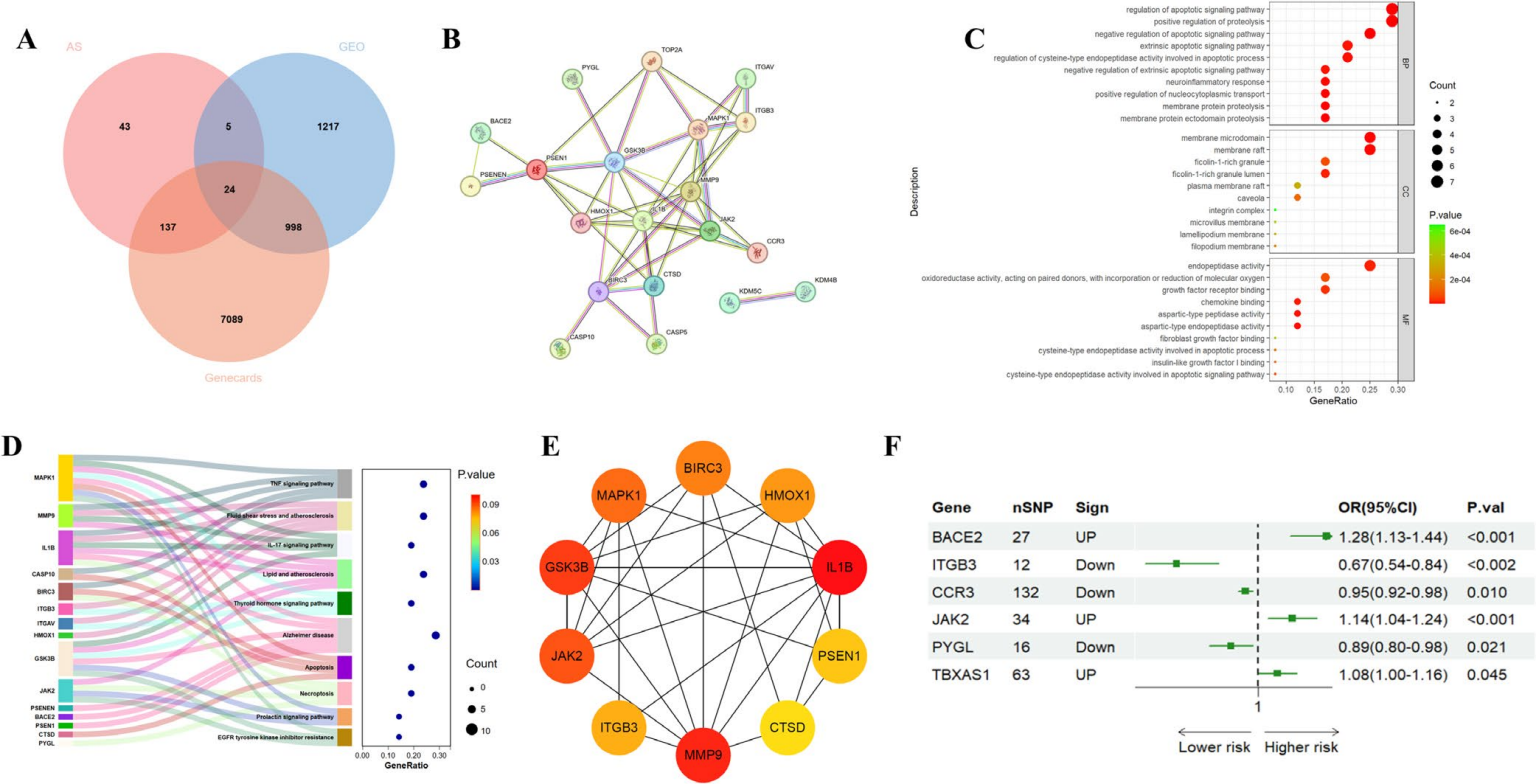
Integrated analysis screening the AS‐associated molecular networks and functional enrichment in Multiple sclerosis. (A) Venn diagram of AS targets overlapping in GeneCards and GEO dataset. (B) PPI network of core overlapping targets. (C) GO enrichment analysis of the overlapping target genes identified, divided into three categories: Including BP, CC, and MF. (D) KEGG enrichment analysis showed the top 10 pathways with the highest count values and identified key genes in these 10 pathways. (E) The top 10 core targets with the highest scores identified by MCC algorithm. (F) MR analysis screening of core targets in overlapping targets. The *x*‐axis represents the odds ratio (OR), with the vertical line indicating the null effect line (OR = 1). Green Square represent the OR values obtained from different MR analysis methods, with horizontal lines denoting the 95% confidence intervals (CI).

A total of 8248 and 2244 MS‐related genes were screened from the GeneCards and GEO databases, respectively. Intersection analysis between MS‐related genes and ARGs identified 24 overlapping genes (Table S7), which were considered potential ARGs‐induced MS‐associated targets (Figure 3A,B). Functional enrichment analysis of these 24 targets indicated that ARGs‐induced MS mechanisms were mainly associated with BP such as neuroinflammatory response, apoptotic signaling pathways, and positive regulation of proteolysis. MF analysis showed enrichment in endopeptidase activity and chemokine binding, while CC analysis suggested predominant localization in membrane rafts, membrane microdomains, and filopodial membranes (Figure 3C). KEGG pathway analysis revealed significant enrichment in the TNF, IL‐17, and prolactin signaling pathways (Figure 3D).

To further identify core ARGs in MS, the MCC algorithm was conducted to identify IL1B, MMP9, GSK3B, JAK2, MAPK1, BIRC3, HMOX1, ITGB3, PSEN1, and CTSD as hub genes (Figure 3E; Table S8). Subsequently, two‐sample MR analysis demonstrated that BACE2, ITGB3, CCR3, JAK2, PYGL, and TBXAS1 exhibited statistically significant causal associations with MS progression (Figure 3F). After excluding JAK2 due to inconsistency with differential expression analysis, ITGB3 was ultimately identified as a robust candidate target mediating ARGs‐induced MS. Further MR analysis focusing on ITGB3 indicated that increased genetically predicted ITGB3 expression was associated with a reduced risk of MS, while sensitivity analyses further supported the robustness of this association (Figure S3C; Table S9).

### 3.6 Biological Effects and Identification of Core ARGs in Inflammatory Bowel Disease

**FIGURE 4:**
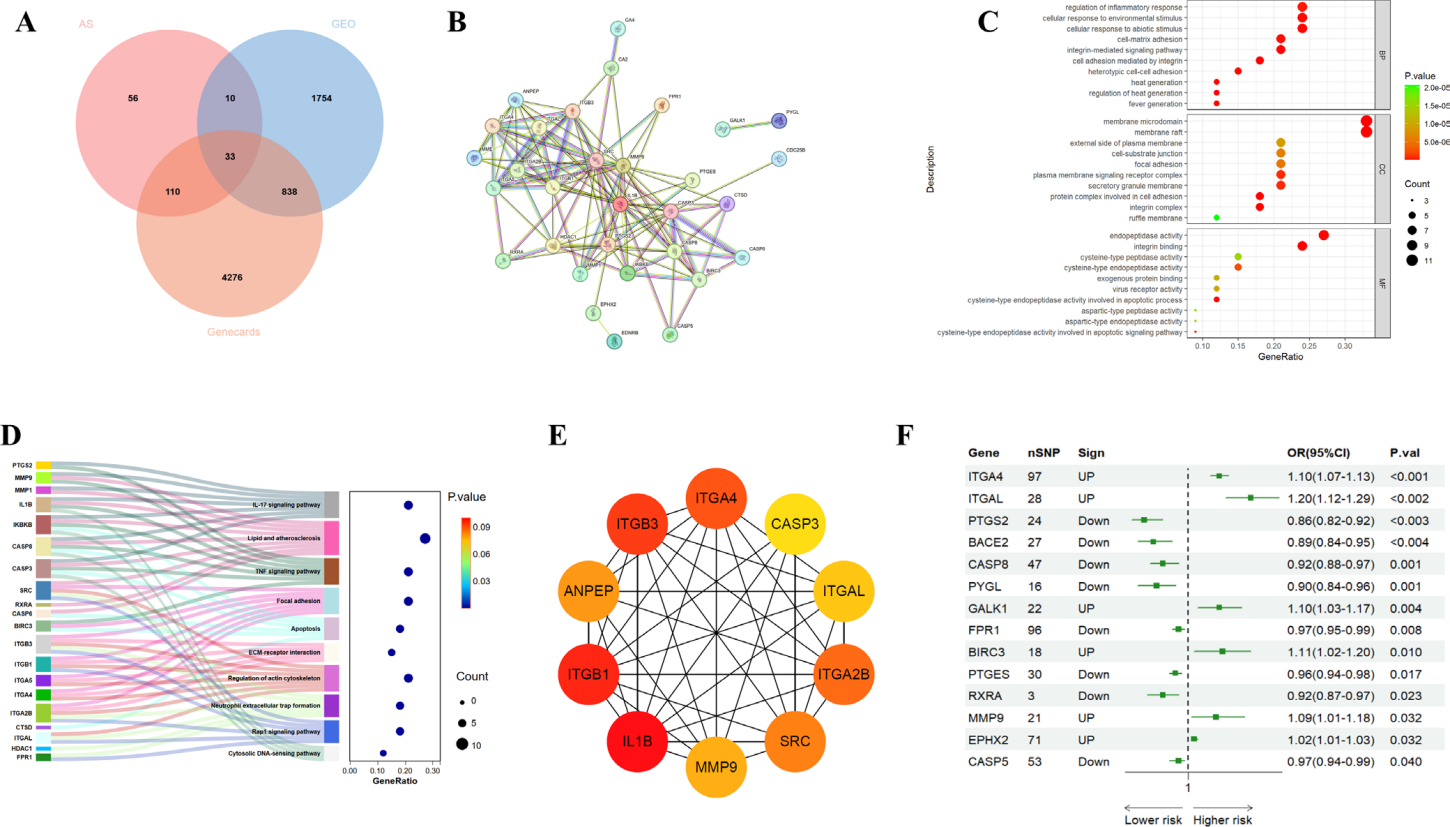
Integrated analysis screening the AS‐associated molecular networks and functional enrichment in inflammatory bowel disease. (A) Venn diagram of AS targets overlapping in GeneCards and GEO dataset. (B) PPI network of core overlapping targets. (C) GO enrichment analysis of the overlapping target genes identified, divided into three categories: Including BP, CC, and MF. (D) KEGG enrichment analysis showed the top 10 pathways with the highest count values and identified key genes in these 10 pathways. (E) The top 10 core targets with the highest scores identified by MCC algorithm. (F) MR analysis screening of core targets in overlapping targets. The *x*‐axis represents the odds ratio (OR), with the vertical line indicating the null effect line (OR = 1). Green squares represent the OR values obtained from different MR analysis methods, with horizontal lines denoting the 95% confidence intervals (CI).

A total of 5257 and 2635 IBD‐related genes were retrieved from the GeneCards and GEO databases, respectively. Intersection analysis between IBD‐related genes and ARGs identified 33 overlapping genes (Table S7), which formed a tightly connected PPI network (Figure 4A,B). Functional enrichment analysis indicated that ARGs‐induced IBD mechanisms were mainly associated with BP such as integrin‐mediated signaling pathways, cellular responses to environmental stimuli, and fever generation. MF analysis showed enrichment in integrin binding and cysteine‐type endopeptidase activity, while CC analysis suggested predominant localization in integrin complexes and focal adhesions (Figure 4C). KEGG pathway analysis revealed significant enrichment in the IL‐17, TNF, and apoptosis signaling pathways (Figure 4D).

The MCC algorithm identified ITGA4, CASP3, ITGAL, ITGA2B, SRC, MMP9, IL1B, ITGB1, ANPEP, and ITGB3 as core ARGs in IBD (Figure 4E; Table S8). Subsequently, two‐sample MR analysis identified several genes exhibiting statistically significant causal associations with IBD progression, among which MMP9 remained consistent across all analyses (Figure 4F). By integrating MCC and MR analyses, MMP9 was ultimately identified as a robust candidate target mediating ARGs‐induced IBD. Further MR analysis focusing on MMP9 indicated that increased genetically predicted MMP9 expression was associated with a higher risk of IBD, while sensitivity analyses further confirmed the stability and reliability of this association (Figure S3D; Table S9).

### 3.7 Biological Effects and Identification of Core ARGs in Hashimoto's Thyroiditis

**FIGURE 5:**
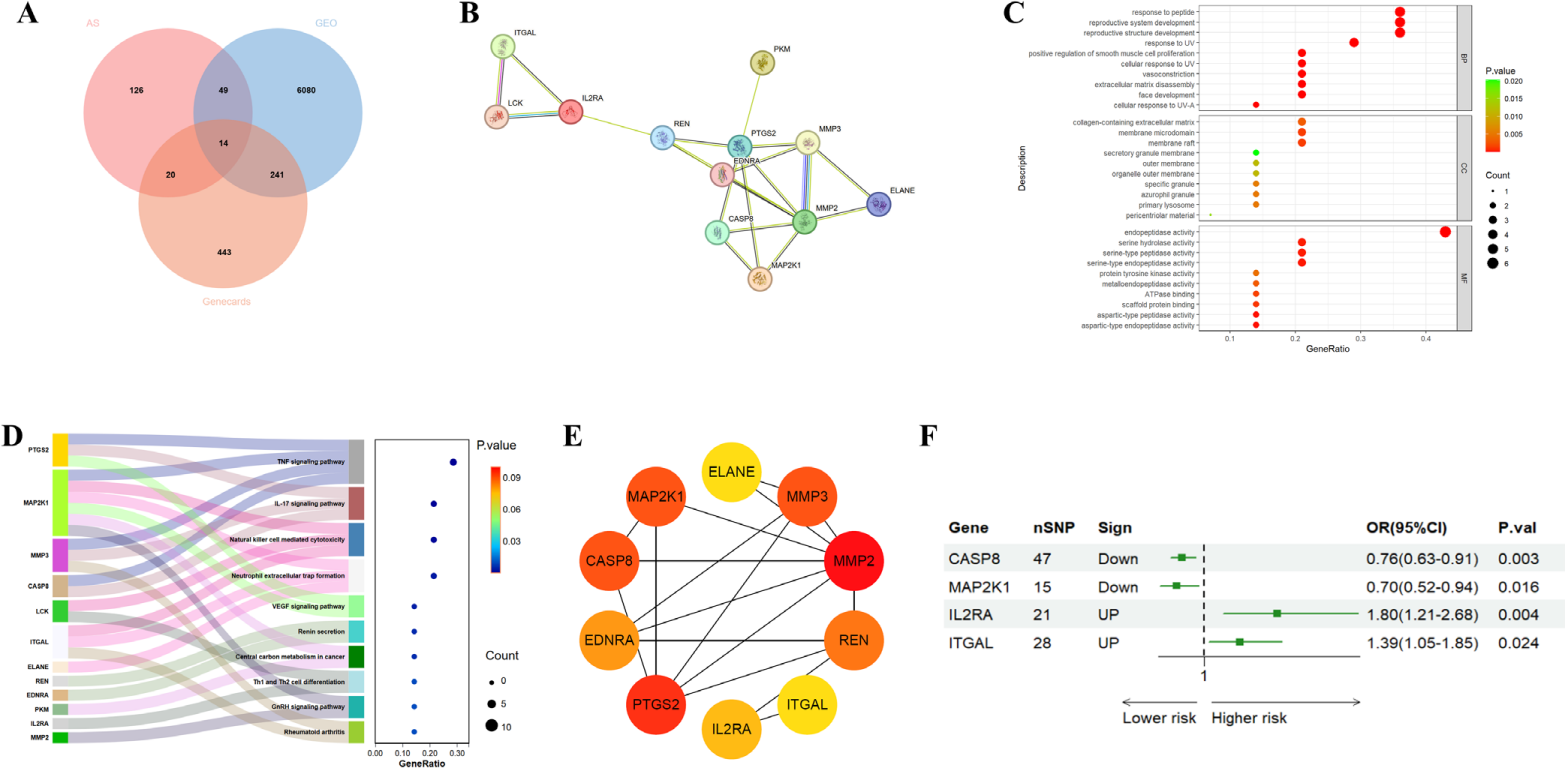
Integrated analysis screening the AS‐associated molecular networks and functional enrichment in Hashimoto's thyroiditis. (A) Venn diagram of AS targets overlapping in GeneCards and GEO dataset. (B) PPI network of core overlapping targets. (C) GO enrichment analysis of the overlapping target genes identified, divided into three categories: including BP, CC, and MF. (D) KEGG enrichment analysis showed the top 10 pathways with the highest count values and identified key genes in these 10 pathways. (E) The top 10 core targets with the highest scores identified by MCC algorithm. (F) MR analysis screening of core targets in overlapping targets. The *x*‐axis represents the odds ratio (OR), with the vertical line indicating the null effect line (OR = 1). Green Square represent the OR values obtained from different MR analysis methods, with horizontal lines denoting the 95% confidence intervals (CI).

A total of 718 and 6384 HT‐related genes were retrieved from the GeneCards and GEO databases, respectively. Intersection analysis between HT‐related genes and ARGs identified 14 overlapping genes (Table S7), which were considered potential ARGs‐induced HT‐associated targets (Figure 5A,B). Functional enrichment analysis indicated that ARGs‐induced HT mechanisms were mainly associated with BP such as response to peptide and extracellular matrix disassembly. MF analysis showed enrichment in regulation of endopeptidase activity and protein tyrosine kinase activity, while CC analysis suggested predominant localization in membrane rafts and secretory granule membranes (Figure 5C). KEGG pathway analysis revealed significant enrichment in the TNF, IL‐17, and natural killer cell‐mediated cytotoxicity pathways (Figure 5D).

To further identify core ARGs in HT, the MCC algorithm was applied to identify ELANE, MMP3, MMP2, REN, ITGAL, IL2RA, PTGS2, EDNRA, CASP8, and MAP2K1 as hub genes (Figure 5E; Table S8). Subsequently, two‐sample MR analysis demonstrated that CASP8, IL2RA, MAP2K1, and ITGAL exhibited statistically significant causal associations with HT progression (Figure 5F). After excluding CASP8 due to inconsistency with differential expression analysis, IL2RA was ultimately identified as a robust candidate target mediating ARGs‐induced HT based on integrative analysis and previous literature (Yao et al. 2024). Further MR analysis focusing on IL2RA indicated that IL2RA acted as the most significant risk factor for HT, and sensitivity analyses further confirmed the stability and reliability of this association (Figure S3E; Table S9).

### 3.8 Biological Effects and Identification of Core ARGs in Type 1 Diabetes

**FIGURE 6:**
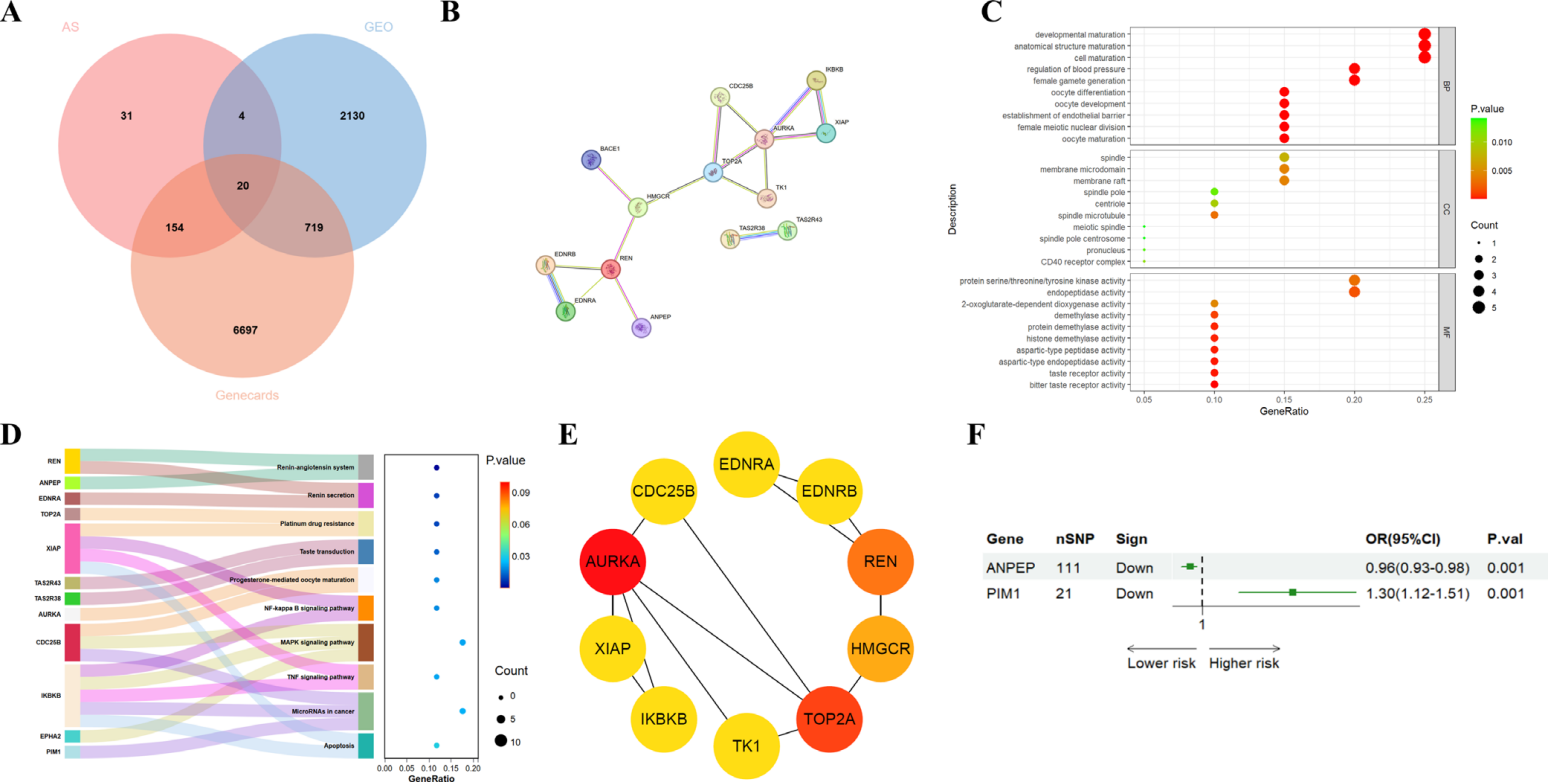
Integrated analysis screening the AS‐associated molecular networks and functional enrichment in type 1 diabetes. (A) Venn diagram of AS targets overlapping in GeneCards and GEO dataset. (B) PPI network of core overlapping targets. (C) GO enrichment analysis of the overlapping target genes identified, divided into three categories: Including BP, CC, and MF. (D) KEGG enrichment analysis showed the top 10 pathways with the highest count values and identified key genes in these 10 pathways. (E) The top 10 core targets with the highest scores identified by MCC algorithm. (F) MR analysis screening of core targets in overlapping targets. The *x*‐axis represents the odds ratio (OR), with the vertical line indicating the null effect line (OR = 1). Green square represent the OR values obtained from different MR analysis methods, with horizontal lines denoting the 95% confidence intervals (CI).

A total of 7590 and 2873 T1D‐related genes were obtained from the GeneCards and GEO databases, respectively. Intersection analysis between T1D‐related genes and ARGs identified 20 overlapping genes, which were considered potential ARGs‐induced T1D‐associated targets (Figure 6A,B). Functional enrichment analysis indicated that ARGs‐induced T1D mechanisms were mainly associated with BP such as cell maturation and endothelial barrier establishment. MF analysis showed enrichment in epigenetic modification activities, while CC analysis suggested predominant localization in membrane rafts and spindles (Figure 6C). KEGG pathway analysis revealed significant enrichment in the NF‐κB, MAPK, and TNF signaling pathways (Figure 6D).

To further identify core ARGs in T1D, the MCC algorithm was applied to identify AURKA, TOP2A, REN, HMGCR, TK1, CDC25B, XIAP, IKBKB, EDNRB, and EDNRA as hub genes (Figure 6E; Table S8). Subsequently, two‐sample MR analysis demonstrated that PIM1 and ANPEP exhibited statistically significant causal associations with T1D progression (Figure 6F). Given the established role of PIM1 in T1D pathogenesis (Smyth et al. 2021), PIM1 was ultimately identified as a robust candidate target mediating ARGs‐induced T1D. Further MR analysis focusing on PIM1 indicated that PIM1 acted as the most significant risk factor for T1D, while sensitivity analyses further supported the robustness of this association (Figure S3F; Table S9).

### 3.9 Biological Overall Effects of ARGs in Autoimmune Diseases

Based on the distinct molecular mechanisms through which AS may contribute to the progression of the six autoimmune diseases, we further investigated the overall biological effects of ARGs in ADs. After integrating and removing duplicated AS‐related targets across all ADs, a total of 87 potential ARGs associated with AD development and progression were identified. PPI network analysis demonstrated extensive interactions among these targets, generating 515 tightly connected edges with an average node degree of 11.8 (*p* < 1.0e‐16), indicating a highly interconnected regulatory network (Figure S4A). Subsequent enrichment analyses were performed to evaluate the overall biological functions of these ARGs in ADs. GO enrichment analysis revealed that these targets were mainly involved in biological processes such as regulation of inflammatory response, canonical NF‐kappaB signal transduction, and positive regulation of neuron apoptotic process (Figure S4B). KEGG pathway analysis demonstrated significant enrichment in apoptosis, TNF signaling, and IL‐17 signaling pathways (Figure S4C). Collectively, these GO and KEGG enrichment results were closely associated with the pathogenic mechanisms of ADs, further highlighting the critical role of ARGs in mediating immune dysregulation and chronic inflammatory responses in autoimmune diseases.

### 3.10 Molecular Docking Analysis

**FIGURE 7:**
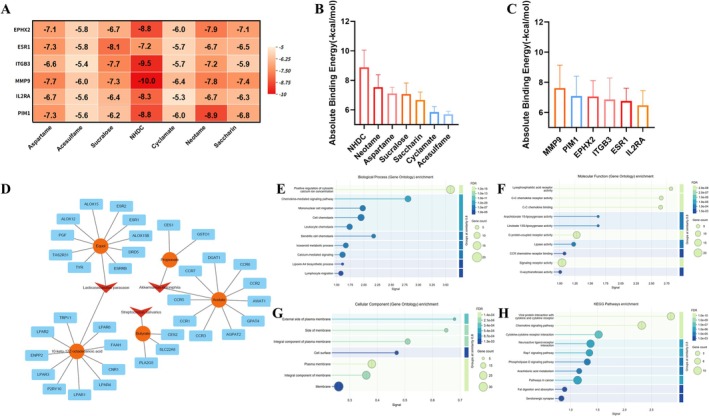
Molecular docking analysis of AS interactions with core AD‐associated targets. (A) Binding affinity heatmap of 7 AS compounds (rows) against 9 core targets (columns), color‐scaled by docking energy (kcal/mol). (B) Ranked average binding energies of 7 AS compounds. Every AS is sorted by mean binding energy. (C) Ranked average binding energies of 6 targets with AS compounds. Targets are sorted by mean binding energy. (D) Protein–protein interaction network constructed using the CheMBLE and SEA databases, integrating three key gut microbiota ( *Akkermansia muciniphila* ; *Lacticaseibacillus paracasei*; *Streptococcus salivarius* ), metabolites, and target genes. Red triangles represent microbial taxa, orange circles denote metabolites, blue rectangles symbolize microbial metabolite targets. (E) Top 10 enriched terms for biological processes, (F) Molecular functions, and (G) Cellular components. (H) Top 10 enriched KEGG pathways. Bubble size corresponds to the number of enriched target genes, while color intensity reflects the false discovery rate (FDR).

To explore potential interactions between AS and the core AARGs identified through bioinformatics analysis, we employed molecular docking, a widely used computational method for predicting potential binding affinities and interaction modes at the molecular level. This approach allows for the assessment of how environmental chemicals, such as AS, may interact with disease‐related proteins, providing preliminary structural insights that can guide further experimental investigation. We performed molecular docking to analyze the interactions between the seven AS and the six identified core targets (Figure S5; Figure 6). Detailed information regarding UniProt accessions, gene symbols, and RCSB IDs for these targets is provided in Table S10. For each protein‐AS pair, docking was performed five times, and the most favorable binding energy was selected for analysis, resulting in a total of 42 measurements (Figure 7A). The overall binding profile of each protein was represented by its average binding energy across all AS molecules. This systematic prioritization provides a structural basis for understanding AS‐related biological effects and highlights high‐priority targets for subsequent mechanistic validation (Figure 7B,C).

### 3.11 Gut Microbiota Analysis

Considering that AS are primarily processed through the gastrointestinal tract, we further explored the potential involvement of gut microbiota in ARGs using microbiota–host gene association analyses. Based on the GutMGene database, three microbial strains sharing host genes with ARGs were identified, including *Akkermansia muciniphila, Lacticaseibacillus paracasei*, and *Streptococcus salivarius* . These strains were associated with the host genes MAPK1, IL1B, and PTGS2, respectively.

Subsequently, five microbiota‐derived metabolites, including acetate, propionate, 10‐keto‐12Z‐octadecenoic acid, equol, and butyrate, were identified from the three strains. After integration of the SEA and ChEMBL databases, removal of duplicate genes, and selection of the top 10 target genes for each metabolite, a microbiota–metabolite–protein interaction network was constructed (Figure 7D). Functional enrichment analysis suggested that these metabolites may be associated with BP related to cytosolic calcium ion regulation, icosanoid metabolism, and G protein‐coupled chemoattractant receptor activity (Figure 7E). MF enrichment further indicated potential involvement in chemokine receptor activity, arachidonate 15‐lipoxygenase activity, and lipase activity (Figure 7F), while CC analysis suggested predominant localization at the plasma membrane and its external surface (Figure 7G). KEGG and GeneMANIA analyses further indicated possible enrichment in pathways related to arachidonic acid metabolism, chemokine signaling, and phospholipase D signaling (Figure 7H; Figure S7). These comprehensive multi‐omics linkages suggest that potential interactions among AS‐associated gut microbiota, microbial metabolites, and host immune‐related signaling pathways.

## 4 Discussion

In the present study, we combined toxicity prediction, Mendelian randomization, network toxicology, molecular docking, and gut microbiota analyses to systematically investigate the potential immunotoxic effects of AS on ADs. Our findings demonstrated the overall immunotoxic effects of AS on AD susceptibility and further identified disease‐specific molecular mechanisms and core targets involved in six autoimmune diseases, including RA, SLE, MS, IBD, HT, and T1D. In addition, gut microbiota analysis supported a potential association among AS exposure, microbial dysbiosis, immune signaling activation, and AD progression. Collectively, these findings highlight the critical role of AS‐driven immune dysregulation, gut microbiota imbalance, and chronic inflammatory activation in the initiation and progression of autoimmune diseases.

RA is a chronic systemic autoimmune disease characterized by persistent synovial inflammation, progressive cartilage destruction, and immune‐mediated joint injury. A previous French prospective cohort study reported that consumption of coffee and artificially sweetened soft drinks was associated with an increased risk of RA (Ascione et al. 2023) (HR = 1.66; 95% CI: 1.12–2.45). Our integrative analysis identified EPHX2 as a potential core target linking AS exposure to RA progression. EPHX2 is involved in the regulation of macrophage‐mediated inflammatory signaling and immune responses in chronic inflammatory diseases (She et al. 2023). In psoriasis models, EPHX2 deficiency aggravates inflammatory cell infiltration and epidermal hyperplasia, supporting its protective immunomodulatory role (Naeem et al. 2024). These findings suggest that AS exposure may promote RA progression by disrupting EPHX2‐mediated immune regulation and enhancing chronic inflammatory activation.

SLE is a complex systemic autoimmune disease characterized by autoantibody production, immune complex deposition, and chronic multi‐organ inflammation. Previous studies have demonstrated that AS may induce immune dysregulation and systemic inflammatory activation through metabolic disturbances and gut microbiota alterations, which are also central mechanisms involved in SLE pathogenesis (Raoul et al. 2022; Sun and Xu 2025). In addition, AS exposure has been reported to influence the progression of Alzheimer's disease through ESR1‐related pathways, which is consistent with our finding (Ge et al. 2025). As a critical mediator of estrogen signaling, ESR1 regulates T‐cell differentiation, B‐cell activation, and cytokine secretion, thereby maintaining immune homeostasis (He et al. 2024). Dysregulated ESR1 signaling has been associated with lupus nephritis and other immune‐mediated inflammatory disorders, while population‐based studies further indicate that ESR1 interacts with genetic and environmental factors to influence SLE susceptibility (Zhou et al. 2017). These findings suggest that AS exposure may accelerate SLE progression by disrupting ESR1‐mediated immune regulation and promoting chronic inflammatory responses.

MS is a chronic autoimmune demyelinating disease of the central nervous system characterized by neuroinflammation, immune‐cell infiltration, and progressive neuronal injury. Previous studies have shown that long‐term AS exposure may induce persistent microglial activation and excessive release of pro‐inflammatory mediators. Moreover, AS‐associated gut microbiota alterations may further influence neuroinflammation and neuroimmune homeostasis (Chen et al. 2022; Wallrapp and Chiu 2024). These combined inflammatory effects may contribute to neuronal degeneration and impaired neuroimmune regulation, thereby increasing susceptibility to MS (Dar 2024). In our study, ITGB3 was identified as a disease‐specific target potentially mediating the association between AS exposure and MS progression. ITGB3 belongs to the integrin family and mediates interactions among immune cells, endothelial cells, and antigen‐presenting cells. Previous studies have demonstrated that dysregulated ITGB3 signaling contributes to endothelial inflammation and NF‐κB‐mediated immune activation, thereby facilitating neuroinflammation and immune‐cell migration into the central nervous system (Li et al. 2024; Luo et al. 2025). Our findings suggest that AS exposure may promote MS progression through disruption of ITGB3‐mediated immune regulation and enhancement of chronic neuroinflammatory responses.

IBD is a chronic immune‐mediated gastrointestinal disorder characterized by epithelial barrier dysfunction, persistent mucosal inflammation, and dysregulated immune responses. Increasing evidence indicates that AS exposure impairs gut microbial homeostasis and epithelial barrier integrity, thereby promoting chronic intestinal inflammation and increased susceptibility to IBD (Guo et al. 2021; Shil et al. 2020). In our study, MMP9 was identified as a potential core target linking AS exposure to IBD progression. As a major extracellular matrix‐degrading enzyme, MMP9 disrupts intestinal epithelial tight junctions through NF‐κB‐dependent inflammatory mechanisms, resulting in increased intestinal permeability and enhanced mucosal immune activation. Elevated MMP9 expression has also been associated with increased infiltration of neutrophils, macrophages, and activated dendritic cells in inflammatory diseases (Al‐Sadi et al. 2021). Furthermore, pharmacological inhibition of MMP9 alleviates inflammatory pathology in psoriasis and arthritis models (Feng et al. 2025). These findings support the hypothesis that AS exposure may aggravate intestinal inflammation and epithelial barrier damage through MMP9‐mediated immune‐inflammatory signaling pathways.

HT is characterized by immune‐mediated thyroid tissue destruction and chronic inflammatory activation associated with loss of immune tolerance. Although direct evidence regarding the adverse effects of AS on HT remains limited, our study identified shared biological targets between AS exposure and HT. Increasing evidence suggests that AS may contribute to autoimmune disorders by disrupting immune homeostasis, altering gut microbiota composition, and promoting chronic inflammation. In the present study, IL2RA was identified as a potential core target linking AS exposure to thyroid autoimmunity. In HT, IL2RA acts as a DNA methylation initiation site that primarily influences the differentiation and function of Tregs in the thyroid gland (Zhang et al. 2021). Genetic variants within the IL2RA locus are strongly associated with autoimmune thyroid disease, MS, and several other autoimmune disorders (Lowe et al. 2007). In addition, aberrant IL2RA expression promotes CD4+ T‐cell expansion and inflammatory activation in immune‐mediated intestinal diseases (Joosse et al. 2021). These findings suggest that AS exposure may impair IL2RA‐mediated immune tolerance mechanisms and thereby promote thyroid autoimmune inflammation.

T1D is a chronic autoimmune disease characterized by T‐cell‐mediated destruction of pancreatic β‐cells and progressive loss of insulin secretion. Previous studies demonstrated that aspartame may interact with insulin and promote the formation of cytotoxic nanostructures through cross‐β assembly, thereby inducing insulin amyloid aggregation, protease resistance, and cellular toxicity (Ansari et al. 2025). These findings suggest a direct interaction between AS and insulin‐related molecular pathways and may represent a potential mechanism underlying ARGs‐induced T1D susceptibility. Our results further emphasized the critical role of PIM1 in this process. PIM1 is a serine/threonine kinase involved in cell survival, metabolism, and immune regulation through STAT3‐ and STAT5‐dependent signaling pathways. Previous studies have demonstrated that PIM1 promotes inflammatory responses and immune‐cell activation in several autoimmune and chronic inflammatory diseases (Chen, Huang, et al. 2025). Moreover, PIM1 contributes to intestinal inflammation and gut homeostasis disruption through regulation of Wnt‐related signaling and epigenetic pathways, thereby potentially aggravating systemic immune activation (Yang et al. 2024). Given the critical role of chronic inflammation and immune‐cell dysregulation in T1D pathogenesis, these findings suggest that AS exposure may promote pancreatic autoimmune injury through PIM1‐mediated inflammatory and microbiota‐associated signaling pathways.

Although these ADs exhibit distinct clinical manifestations, they share several common immunopathological features, including chronic innate immune activation, Th17/Treg imbalance, excessive cytokine production, epithelial barrier dysfunction, and persistent activation of TNF‐α, IL‐17, NF‐κB, and PI3K‐Akt signaling pathways (Park and Ciofani 2025). Our enrichment analyses demonstrated that AS‐associated targets were significantly enriched in these inflammatory and immune‐regulatory pathways, suggesting that AS may contribute to multiple ADs through convergent mechanisms.

Notably, accumulating evidence suggests that AS‐associated gut microbiota dysbiosis may represent an important bridge linking environmental exposure and autoimmune dysregulation (Raoul et al. 2022; Zhou et al. 2023). We speculate that AS exposure may promote the progression of ADs by altering the abundance of *Akkermansia muciniphila, Lacticaseibacillus paracasei and Streptococcus salivarius*. Previous studies show depletion of short‐chain fatty acid‐producing bacteria such as *A. muciniphila* impairs Treg differentiation and promotes Th17‐mediated inflammation. Meanwhile, reduced abundance of *A. muciniphila* has also been associated with impaired mucus barrier integrity and aggravated systemic inflammation in multiple autoimmune diseases (Ansaldo et al. 2019; Codoñer et al. 2018). Barrier disruption may further facilitate bacterial translocation and chronic activation of macrophages and dendritic cells, thereby forming a vicious cycle of gut dysbiosis and persistent immune‐inflammatory activation (Effendi et al. 2022). Similarly, decreased abundance of *L.paracasei* and *S.salivarius* may aggravate intestinal dysbiosis, oxidative stress, and inflammatory responses through impaired immune regulation and epithelial barrier repair (Dehghani et al. 2022; Ma, Wu, et al. 2025).

Importantly, our network toxicology and microbiota analyses converged on several common biological processes, particularly inflammatory signaling, epithelial barrier dysfunction, oxidative stress, and arachidonic acid metabolism. For example, EPHX2 participates in arachidonic acid metabolism and inflammatory lipid mediator regulation, while MMP9 contributes to intestinal barrier disruption and leukocyte infiltration. Simultaneously, AS‐associated depletion of beneficial bacteria such as *A.muciniphila* and *L. paracasei* may further aggravate epithelial permeability and inflammatory cytokine release, thereby amplifying activation of TNF, IL‐17, and NF‐κB signaling pathways (Ghotaslou et al. 2023; Chen, Zhang, et al. 2025). These findings suggest that AS‐associated gut dysbiosis may synergize with host inflammatory signaling networks identified through network toxicology analysis. Mechanistically, we suggest excessive AS exposure may initially alter gut microbial ecology and intestinal barrier integrity, resulting in enhanced microbial antigen exposure and oxidative stress. These alterations subsequently activate immune‐related pathways, including TNF, IL‐17, PI3K‐Akt, and focal adhesion signaling, ultimately promoting chronic immune activation and autoimmune tissue injury, which may explain why AS exposure exhibits broad immunotoxic potential across multiple autoimmune disorders despite their heterogeneous clinical phenotypes.

To our knowledge, this is the first study to comprehensively investigate the potential mechanisms linking AS exposure and ADs using an integrated framework combining network toxicology, Mendelian randomization, molecular docking, and gut microbiota analysis. Nevertheless, several limitations should be acknowledged. First, the collective analysis of the seven artificial sweeteners may have overlooked the toxicological heterogeneity among individual sweeteners, thereby limiting the precise interpretation and potential clinical translational value of the findings. Second, relatively relaxed SNP screening criteria were applied to comprehensively identify ARGs, and residual pleiotropy could not be completely excluded. Third, molecular docking reflects relatively static protein‐ligand interactions and may not fully represent dynamic physiological binding processes. Finally, the gut microbiota analysis was primarily based on database association analyses and bioinformatics prediction, and therefore the inferred mechanistic relationships should be interpreted cautiously. Despite these limitations, our findings provide a comprehensive multi‐omics framework for understanding the immunological mechanisms linking AS exposure and autoimmune diseases and identify several promising targets for future experimental and clinical validation.

Despite these limitations, our study provides a comprehensive multi‐omics framework for understanding the potential immunological mechanisms linking AS exposure and autoimmune diseases and identifies several promising targets for future research. Future studies should focus on: (1) Evaluating the compound‐specific exposure and distinct immunotoxic effects of individual artificial sweeteners in ADs (2) Larger GWAS datasets and advanced MR approaches, including multivariable MR and MR‐PRESSO, are needed to improve causal inference and reduce potential pleiotropic bias. (3) Molecular dynamics simulations and binding free‐energy analyses should be performed to further evaluate the stability and dynamic behavior of the identified protein‐ligand interactions. (4) Future investigations should validate the identified hub targets and pathways through in vitro experiments, including qPCR, Western blotting, and CRISPR knockout in AS‐exposed immune cells, as well as in vivo animal models evaluating immune‐inflammatory responses following AS exposure. (5) Integrating metagenomic sequencing, metabolomics, fecal microbiota transplantation, and gut‐immune axis investigations will be essential to validate the role of AS‐associated microbial alterations in immune dysregulation and AD progression.

## 5 Conclusion

In this study, we systematically investigated key regulatory genes and associated biological pathways potentially involved in ADs pathogenesis related to AS exposure using a multi‐omics approach. Several core targets including EPHX2, ESR1, ITGB3, MMP9, IL2RA, and PIM1, were identified, providing mechanistic insights into AS‐related immune dysregulation. These findings underscore the potential role of AS exposure in modulating immune pathways relevant to ADs. However, the present study does not establish direct causal effects of AS consumption on disease risk. Future research integrating well‐designed clinical cohort studies, along with in vitro and in vivo experiments, is warranted to validate the observed associations, clarify potential exposure‐response relationships, and further elucidate the contribution of AS as a possible environmental risk factor in autoimmune pathogenesis.

## Author Contributions

**Yupei Liu:** writing – review and editing, writing – original draft, investigation, conceptualization, formal analysis. **Jizhen Huang:** writing – original draft, writing – review and editing, software. **Xue Hu:** validation, formal analysis, data curation. **Shan Tian:** methodology, project administration, investigation. **Jiao Li:** investigation, formal analysis, software. **Shiyun Tan:** methodology, supervision, writing – review and editing. **Weiguo Dong:** supervision, writing – review and editing, funding acquisition.

## Funding

This study was funded by the National Natural Science Foundation of China (Nos. 82370542, 82400613).

## Ethics Statement

This study adhered to the ethical guidelines outlined in the Declaration of Helsinki. Since all datasets used were from public, de‐identified sources, institutional review board approval and informed consent from individual participants were not required.

## Consent

The authors have nothing to report.

## Conflicts of Interest

The authors declare no conflicts of interest.

## Supporting information


**Figure S1.** Normalization results of GEO datasets. (A) GSE93272 (RA); (B) GSE50772 (SLE); (C) GSE21942 (MS); (D) GSE3365 (IBD); (E) GSE138198 (HT); (F) GSE156035 (T1D).

**Figure S2.** Overall effects of AS on ADs and identification of AS‐related targets. (A) The consumption of artificial sweeteners in daily life. (B) Molecular structures of seven commonly used AS. (C) Heatmap of the predicted toxicities of seven AS compounds based on the ProTox‐II database. (D) Scatter plots, funnel plots (E), and leave‐one‐out plots (F) of the two‐sample MR analysis results (Intake of artificial sweetener added to cereal on ADs). (G) Protein–protein interaction (PPI) network of 209 targets constructed using STRING database and Cytoscape software. (H) Gene Ontology (GO) enrichment of AS targets. Top 10 terms per category (Biological Process: BP, Cellular Component: CC, Molecular Function: MF) are shown. (I) Kyoto Encyclopedia of Genes and Genomes (KEGG) pathway analysis. Top 10 enriched pathways ranked by *p*‐value, color indicates −log10 (*p*‐value).

**Figure S3.** Mendelian Randomization Analysis Between Hub ARGs and AD. (A) Scatter plots, funnel plots, and leave‐one‐out plots of the two‐sample MR analysis results (EPHX2 on RA). (B) Scatter plots, funnel plots, and leave‐one‐out plots of the two‐sample MR analysis results (ESR1 on SLE). (C) Scatter plots, funnel plots, and leave‐one‐out plots of the two‐sample MR analysis results (ITGB3 on MS). (D) Scatter plots, funnel plots, and leave‐one‐out plots of the two‐sample MR analysis results (MMP9 on IBD). (E) Scatter plots, funnel plots, and leave‐one‐out plots of the two‐sample MR analysis results (IL2RA on HT). (F) Scatter plots, funnel plots, and leave‐one‐out plots of the two‐sample MR analysis results (PIM1 on T1D).

**Figure S4.** The Overall Effects of ARGs in Autoimmune diseases. (A) Protein–protein interaction (PPI) network of 87 AARGs constructed using STRING database and Cytoscape software. (B) Gene Ontology (GO) enrichment of AARGs. Top 10 terms per category (Biological Process: BP, Cellular Component: CC, Molecular Function: MF) are shown. (C) Kyoto Encyclopedia of Genes and Genomes (KEGG) pathway analysis. Top 10 enriched pathways ranked by *p*‐value; Circle size represents gene count, color indicates −log10 (*p*‐value).

**Figure S5.** Detailed visualization of molecular docking interactions between EPHX2 (A), ESR1 (B), ITGB3 (C), and seven artificial sweeteners.

**Figure S6.** Detailed visualization of molecular docking interactions between MMP9 (A), IL2RA (B), PIM1 (C), and seven artificial sweeteners.

**Figure S7.** GNEMAIN analysis results of targets predicted from microbial metabolites.

**Table S1.** Detailed information of datasets used in Mendelian randomization analysis.
**Table S2.** Detailed information of SNPs data of AS‐related traits and ADs.
**Table S3.** Detailed information on commonly used artificial sweeteners.
**Table S4.** Detailed information of GEO datasets for six Ads used in this study.
**Table S5.** Detailed information of SNPs data of ARGs and six ADs.
**Table S6.** Detailed information on commonly used artificial sweeteners realted genes.
**Table S7.** Intersection genes of AS and six ADs differentially expressed genes.
**Table S8.** MCC ranking, Degree, Betweenness centrality, and Closeness centrality of the top 10 AS‐related genes identified by the MCC algorithm in each autoimmune disease.
**Table S9.** Detailed results of five MR analysis and sensitivity analysis.
**Table S10.** Detailed information of proteins for molecular docking.

## Data Availability

The data that supports the findings of this study are available in the Supporting Information of this article.
