# Supplementary figures and images for: Artificial Sweeteners and Autoimmune Diseases: Insights From Integrative Bioinformatics and Mendelian Randomization
Source: Food Sci Nutr. 2026 Jun 18;14(6):e72032. doi: 10.1002/fsn3.72032 (PMC13277747; doi:10.1002/fsn3.72032)

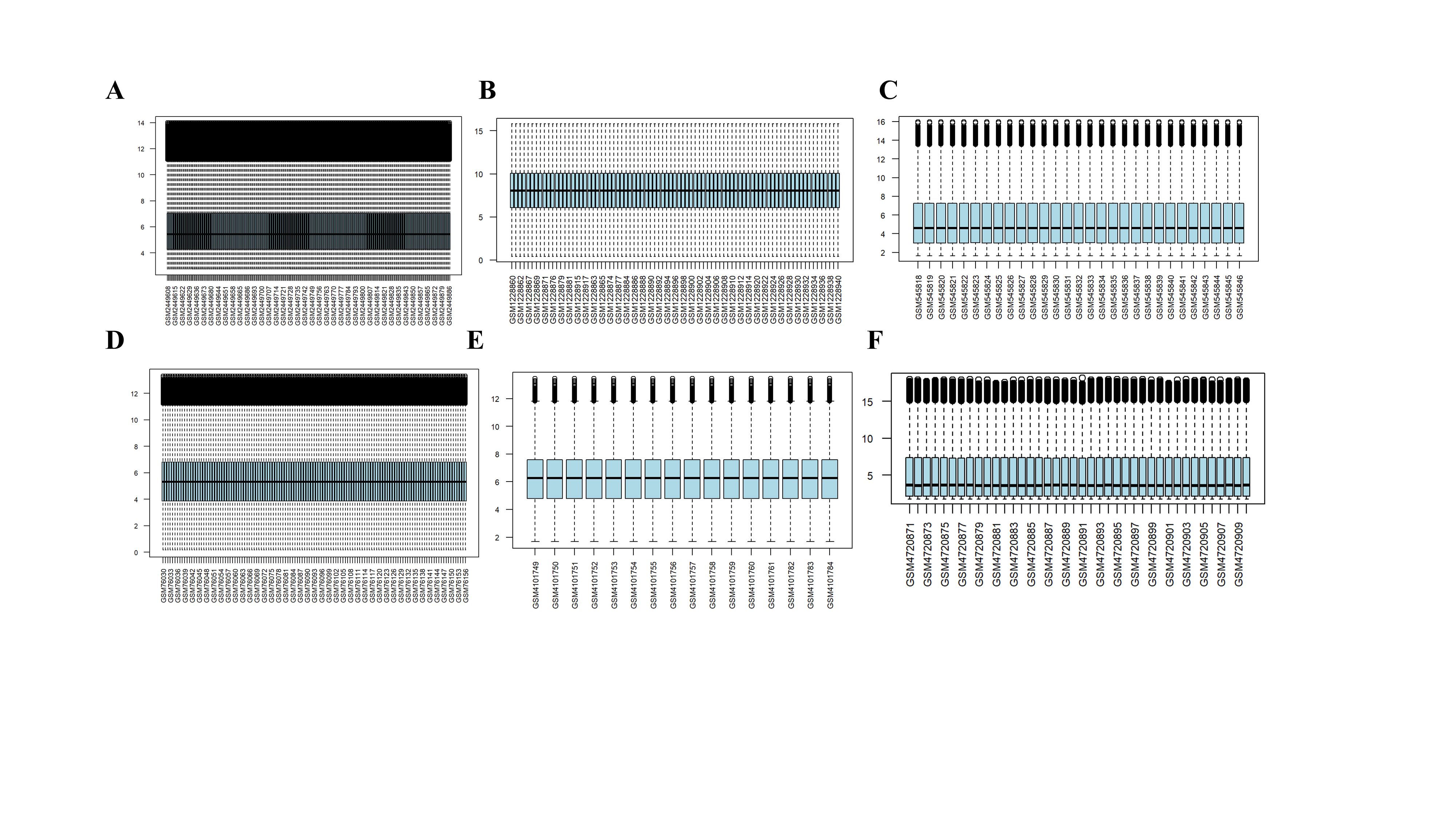

Supplement: Supplementary file 1 — Figure S1. Normalization results of GEO datasets. (A) GSE93272 (RA); (B) GSE50772 (SLE); (C) GSE21942 (MS); (D) GSE3365 (IBD); (E) GSE138198 (HT); (F) GSE156035 (T1D). [file FSN3-14-e72032-s004.tif]

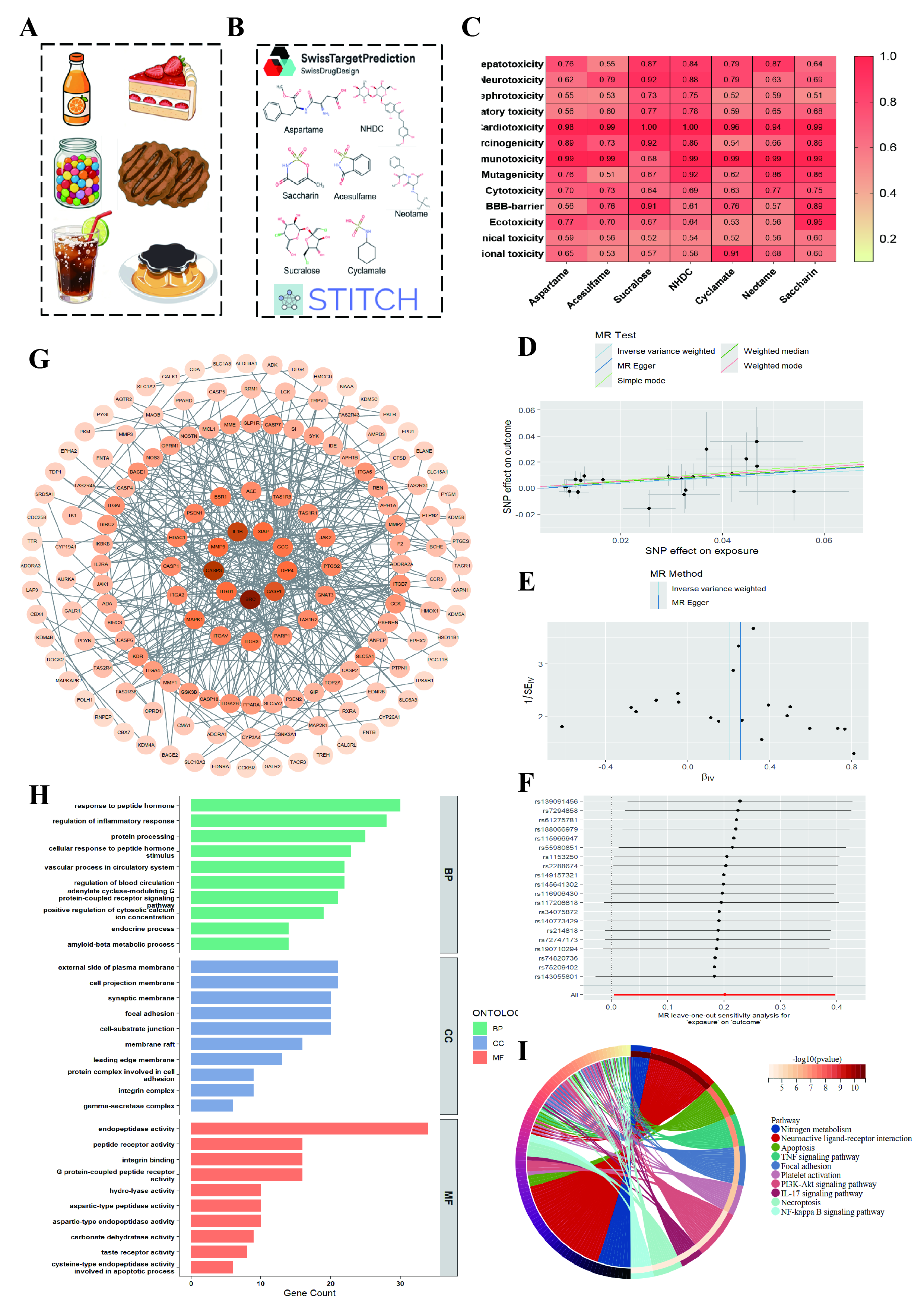

Supplement: Supplementary file 2 — Figure S2. Overall effects of AS on ADs and identification of AS‐related targets. (A) The consumption of artificial sweeteners in daily life. (B) Molecular structures of seven commonly used AS. (C) Heatmap of the predicted toxicities of seven AS compounds based on the ProTox‐II database. (D) Scatter plots, funnel plots (E), and leave‐one‐out plots (F) of the two‐sample MR analysis results (Intake of artificial sweetener added to cereal on ADs). (G) Protein–protein interaction (PPI) network of 209 targets constructed using STRING database and Cytoscape software. (H) Gene Ontology (GO) enrichment of AS targets. Top 10 terms per category (Biological Process: BP, Cellular Component: CC, Molecular Function: MF) are shown. (I) Kyoto Encyclopedia of Genes and Genomes (KEGG) pathway analysis. Top 10 enriched pathways ranked by p‐value, color indicates −log10 (p‐value). [file FSN3-14-e72032-s002.tif]

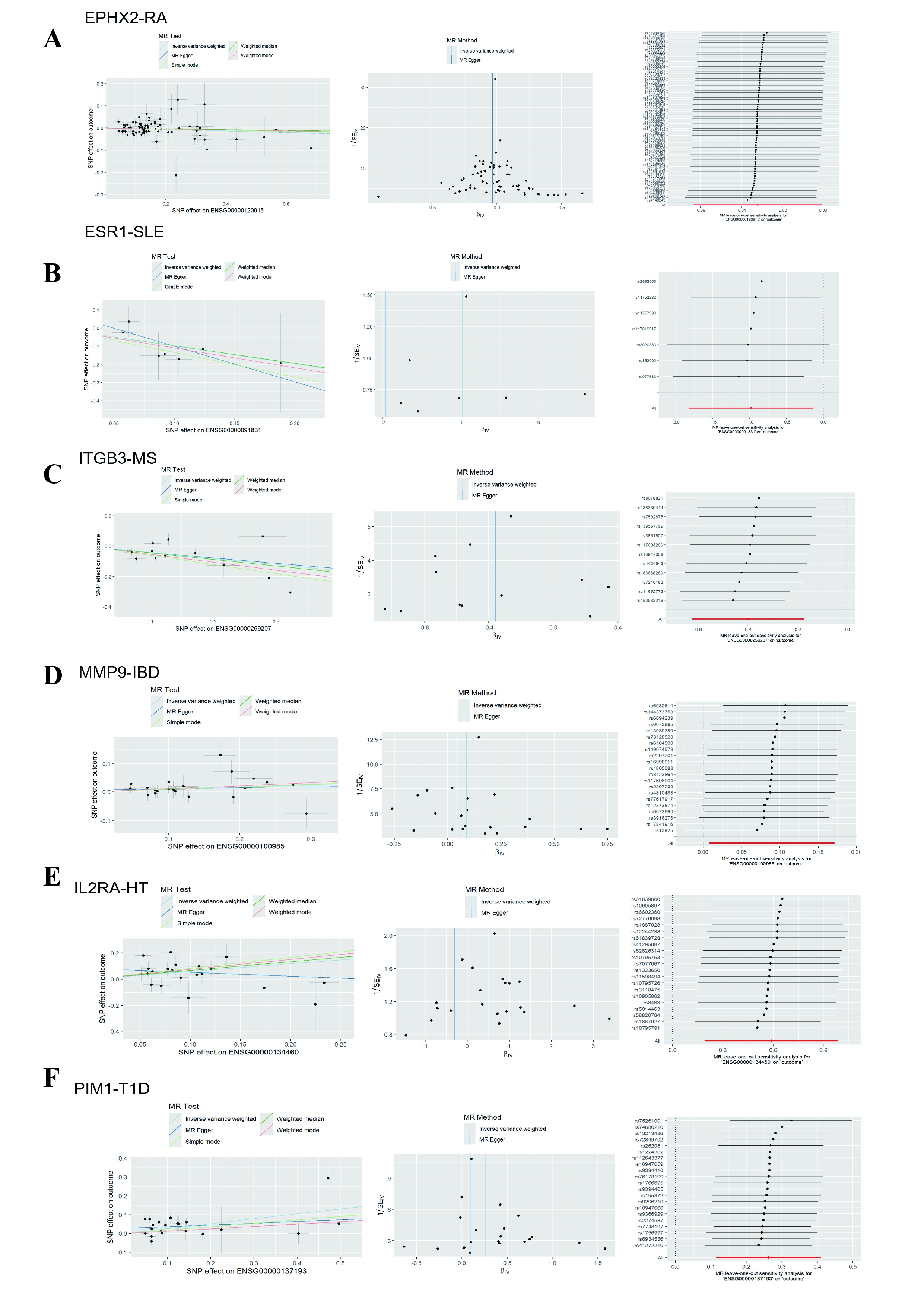

Supplement: Supplementary file 3 — Figure S3. Mendelian Randomization Analysis Between Hub ARGs and AD. (A) Scatter plots, funnel plots, and leave‐one‐out plots of the two‐sample MR analysis results (EPHX2 on RA). (B) Scatter plots, funnel plots, and leave‐one‐out plots of the two‐sample MR analysis results (ESR1 on SLE). (C) Scatter plots, funnel plots, and leave‐one‐out plots of the two‐sample MR analysis results (ITGB3 on MS). (D) Scatter plots, funnel plots, and leave‐one‐out plots of the two‐sample MR analysis results (MMP9 on IBD). (E) Scatter plots, funnel plots, and leave‐one‐out plots of the two‐sample MR analysis results (IL2RA on HT). (F) Scatter plots, funnel plots, and leave‐one‐out plots of the two‐sample MR analysis results (PIM1 on T1D). [file FSN3-14-e72032-s007.tif]

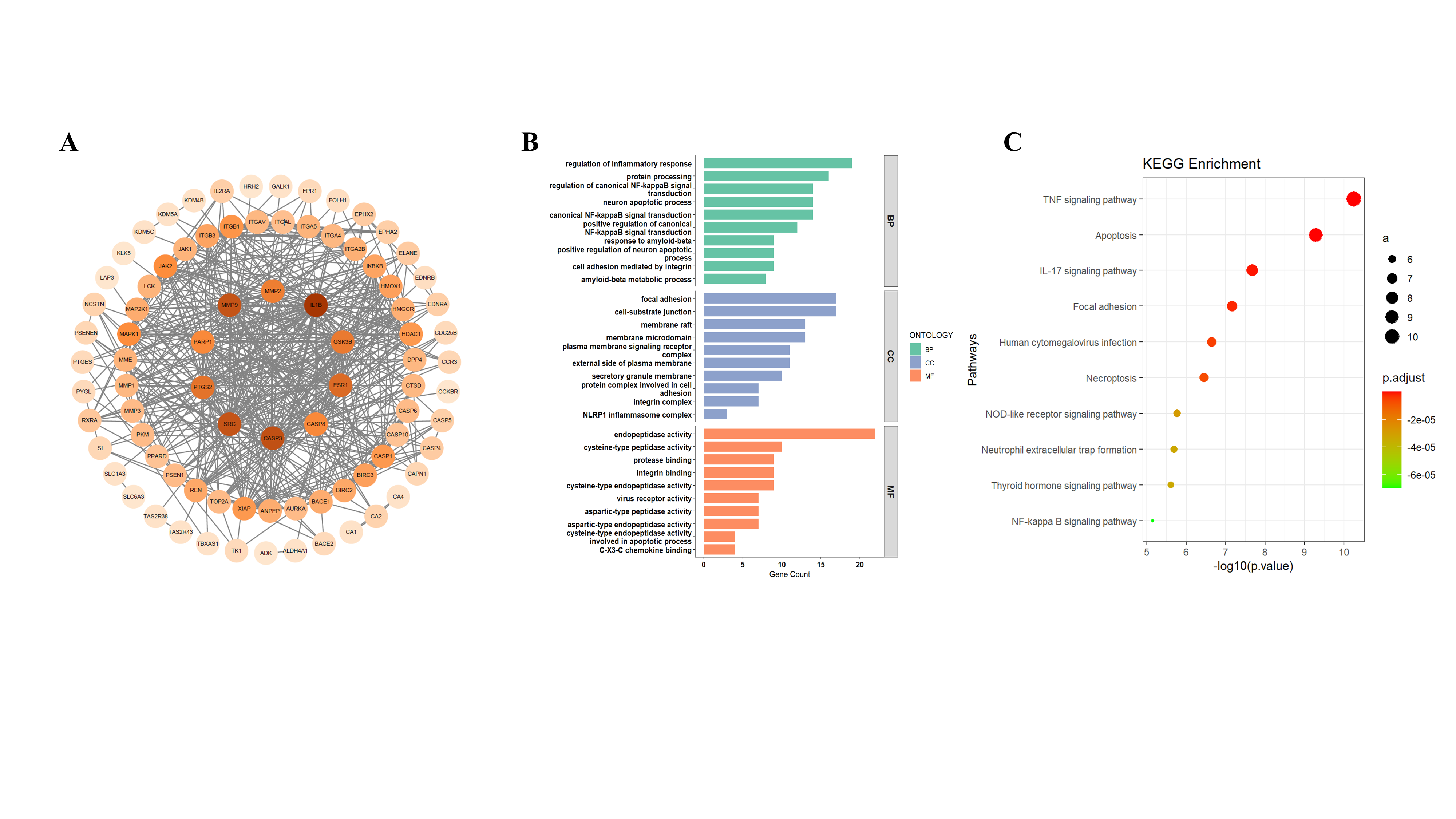

Supplement: Supplementary file 4 — Figure S4. The Overall Effects of ARGs in Autoimmune diseases. (A) Protein–protein interaction (PPI) network of 87 AARGs constructed using STRING database and Cytoscape software. (B) Gene Ontology (GO) enrichment of AARGs. Top 10 terms per category (Biological Process: BP, Cellular Component: CC, Molecular Function: MF) are shown. (C) Kyoto Encyclopedia of Genes and Genomes (KEGG) pathway analysis. Top 10 enriched pathways ranked by p‐value; Circle size represents gene count, color indicates −log10 (p‐value). [file FSN3-14-e72032-s001.tif]

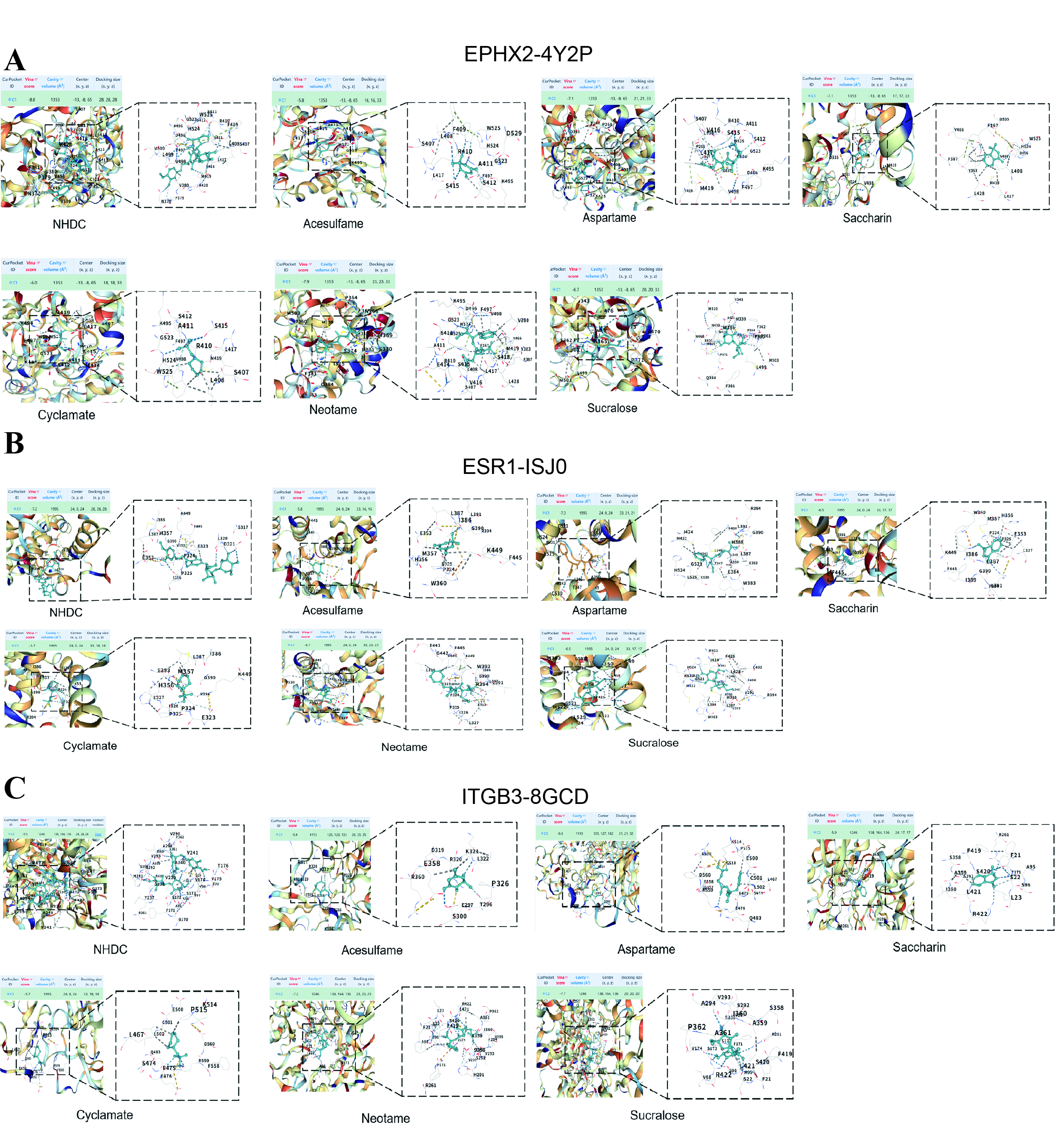

Supplement: Supplementary file 5 — Figure S5. Detailed visualization of molecular docking interactions between EPHX2 (A), ESR1 (B), ITGB3 (C), and seven artificial sweeteners. [file FSN3-14-e72032-s005.tif]

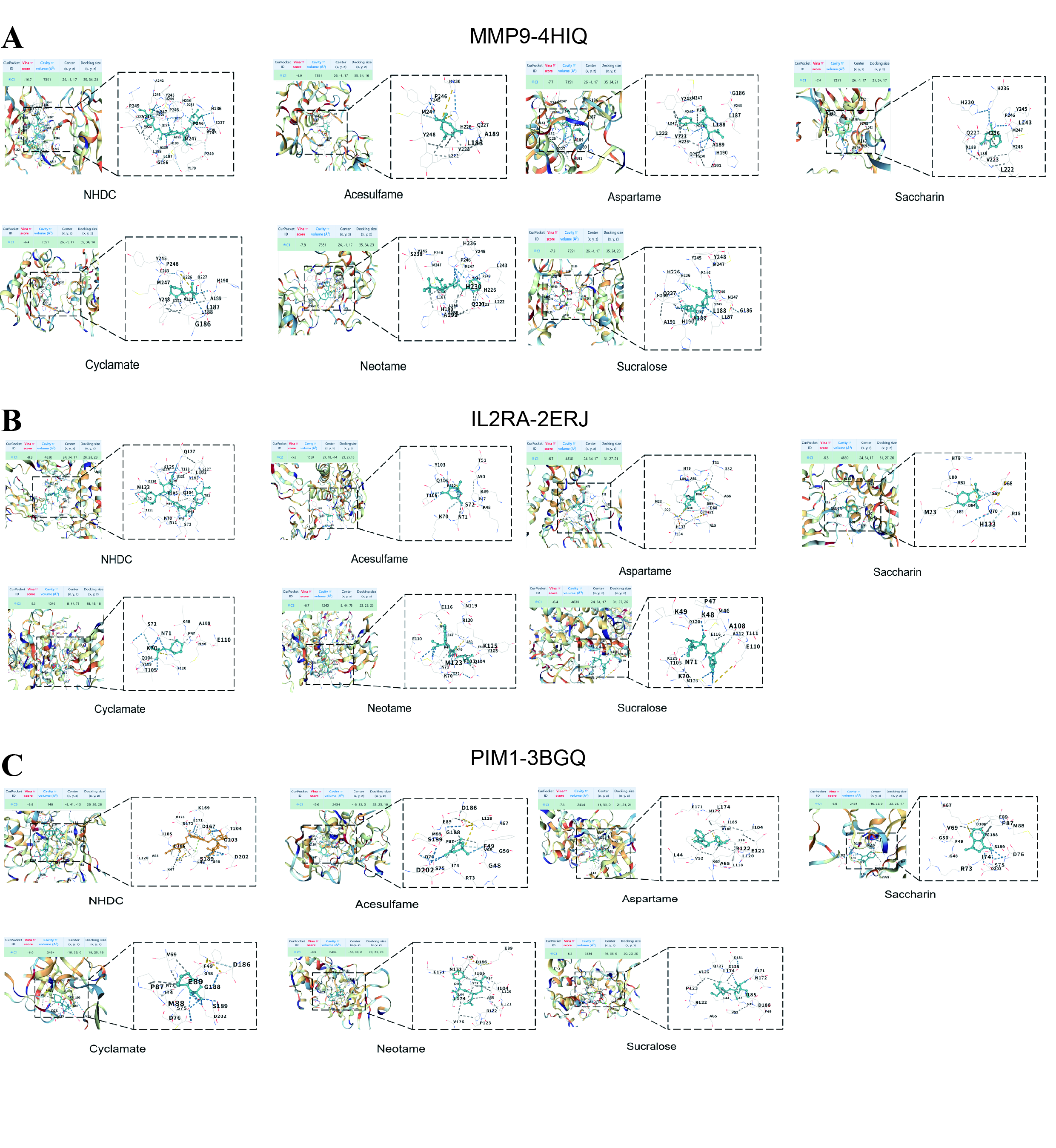

Supplement: Supplementary file 6 — Figure S6. Detailed visualization of molecular docking interactions between MMP9 (A), IL2RA (B), PIM1 (C), and seven artificial sweeteners. [file FSN3-14-e72032-s006.tif]

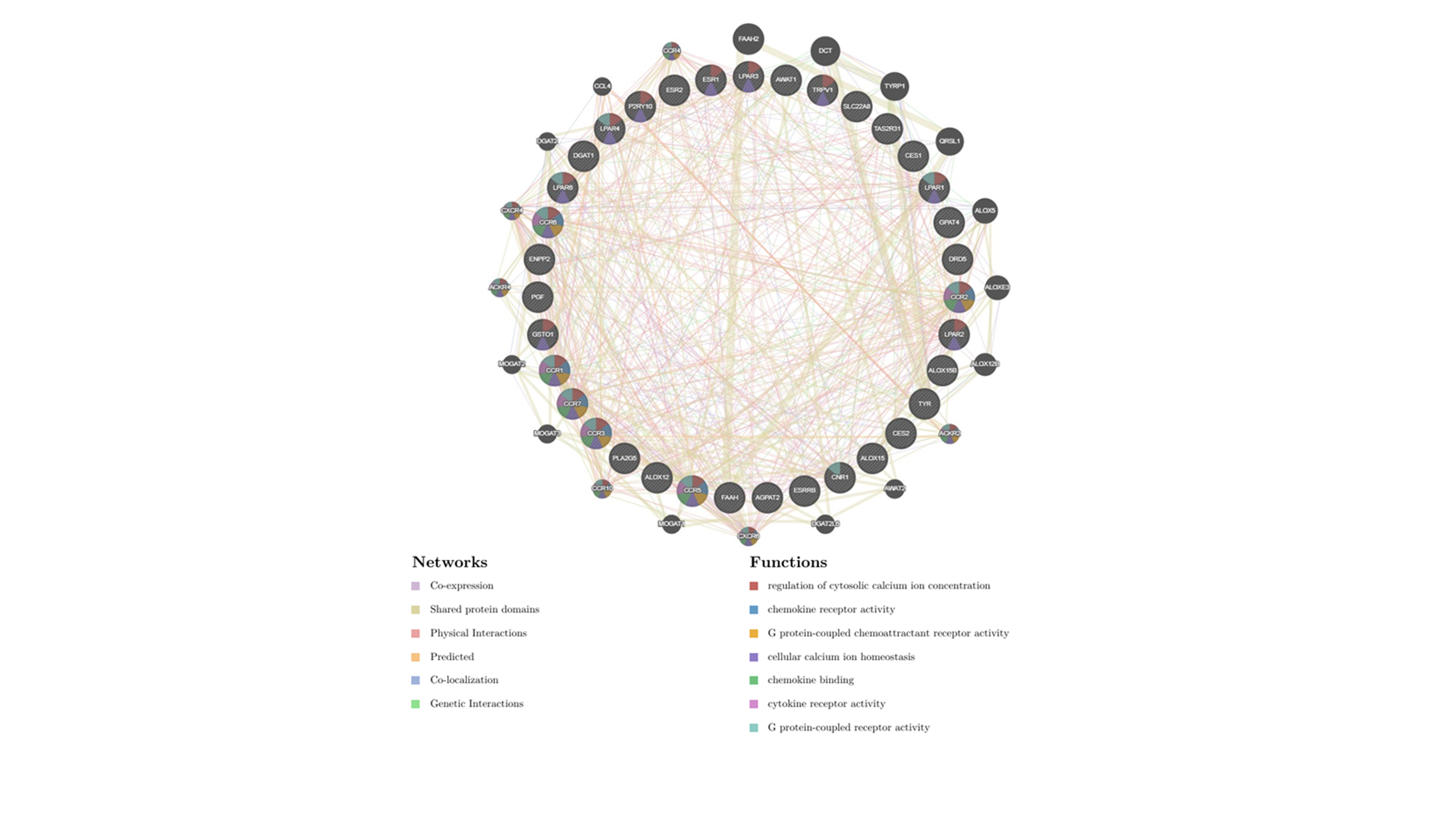

Supplement: Supplementary file 7 — Figure S7. GNEMAIN analysis results of targets predicted from microbial metabolites. [file FSN3-14-e72032-s008.tif]
